# The Role of Kainate Receptors in the Pathophysiology of Hypoxia-Induced Seizures in the Neonatal Mouse

**DOI:** 10.1038/s41598-018-24722-3

**Published:** 2018-05-04

**Authors:** Denise K. Grosenbaugh, Brittany M. Ross, Pravin Wagley, Santina A. Zanelli

**Affiliations:** 1 Department of Neurology, University of Virginia, Charlottesville, Virginia 22908 USA; 2 Department of Pediatrics, University of Virginia, Charlottesville, Virginia 22908 USA

## Abstract

Kainate receptors (KARs) are glutamate receptors with peak expression during late embryonic and early postnatal periods. Altered KAR-mediated neurotransmission and subunit expression are observed in several brain disorders, including epilepsy. Here, we examined the role of KARs in regulating seizures in neonatal C57BL/6 mice exposed to a hypoxic insult. We found that knockout of the GluK2 subunit, or blockade of KARs by UBP310 reduced seizure susceptibility during the period of reoxygenation. Following the hypoxic insult, we observed an increase in excitatory neurotransmission in hippocampal CA3 pyramidal cells, which was blocked by treatment with UBP310 prior to hypoxia. Similarly, we observed increased excitatory neurotransmission in CA3 pyramidal cells in an *in vitro* hippocampal slice model of hypoxic-ischemia. This increase was absent in slices from GluK2^−/−^ mice and in slices treated with UBP310, suggesting that KARs regulate, at least in part, excitatory synaptic neurotransmission following *in vivo* hypoxia in neonatal mice. Data from these hypoxia models demonstrate that KARs, specifically those containing the GluK2 subunit, contribute to alterations in excitatory neurotransmission and seizure susceptibility, particularly during the reoxygenation period, in neonatal mice. Therapies targeting KARs may prove successful in treatment of neonates affected by hypoxic seizures.

## Introduction

Seizures are common in the neonatal period occurring in 1–3 per 1,000 term live births and with incidences as much as 10-fold higher in preterm infants^1,2^. Hypoxia-ischemia is the most common cause of seizures in neonates, accounting for 40–60% of cases^2–5^. Exposure to seizures at this stage of brain development has been linked to an increased risk of cognitive impairments and cerebral palsy as well as a nearly 25% increase in the risk of epilepsy later in life^6–8^. Despite the scope of this problem, successful treatment of neonatal seizures remains a challenge as 40–50% of seizures prove refractory to currently available anti-seizure drugs^9,10^. Additionally, some first-line therapeutic agents such as phenobarbital may interfere and disrupt normal brain development^11,12^ emphasizing the need for novel treatment strategies in the neonatal population. A better understanding of the mechanisms underlying seizure generation after a hypoxic insult is required to develop safer and more effective therapeutic options for neonates with seizures.

Kainate receptors (KARs) are ionotropic glutamate receptors that contribute to fast excitatory neurotransmission and have also been reported to mediate neurotransmission through metabotropic signaling cascades^13,14^. KARs are widely distributed throughout the hippocampus, where they form tetrameric receptor complexes comprised of GluK1–5 subunits, with peak expression occurring during the late embryonic and early postnatal period^15,16^. Through all phases of development, hippocampal CA3 pyramidal cells exhibit robust expression of KAR subunits (GluK2, GluK4, and GluK5), with the heteromeric GluK2/5 receptor combination being most predominant^17,18^. These receptors play an important role in the regulation of excitatory neurotransmission through both pre- and post-synaptic mechanisms in the CA3 region of the hippocampus. Postsynaptic KAR-mediated events are small in amplitude but display slow decay kinetics allowing for temporal summation and an increase in the depolarization envelope^19–21^. KARs localized to presynaptic mossy fibers regulate neurotransmission in a bidirectional manner and also contribute to the frequency-dependent short-term synaptic plasticity characteristic of the mossy fiber – CA3 synapse^17,22–27^.

KARs have been implicated in the pathophysiology of several brain disorders, including epilepsy^28–33^. Alterations in KAR subunit expression have been reported in both animal models of epilepsy and in clinical studies of human temporal lobe epilepsy^34–38^. Despite increasing knowledge of KAR ontogeny and synaptic localization, whether the robust expression of KARs in the neonatal brain contributes to the seizures associated with a hypoxic-insult remains unknown.

The goal of this study was to determine if KARs contribute to the pathophysiology of hypoxia-induced seizures in the neonatal mouse. We hypothesized that KARs, specifically those comprised of the GluK2 subunit, increase seizure susceptibility in the neonatal mouse. Through both genetic and pharmacological manipulation of KARs, we report that neonatal GluK2^−/−^ mice are significantly less susceptible to hypoxia-induced seizures, thus confirming our hypothesis. Further, the increase in excitatory synaptic transmission observed in hippocampal CA3 pyramidal neurons is absent in GluK2^−/−^ mice or in mice treated with a KAR antagonist prior to hypoxia. Results from this study provide the first evidence supporting the role of KARs in modulating response to hypoxia and hypoxia-induced seizures in the neonatal mouse hippocampus. Because hypoxic insults are a major cause of seizures in the neonatal population, the development of therapeutic agents targeting KARs may provide a novel opportunity for age appropriate and mechanism-based treatment.

## Results

### Neonatal GluK2^−/−^ mice are less susceptible to seizures during the reoxygenation period following hypoxia

**Figure 1:**
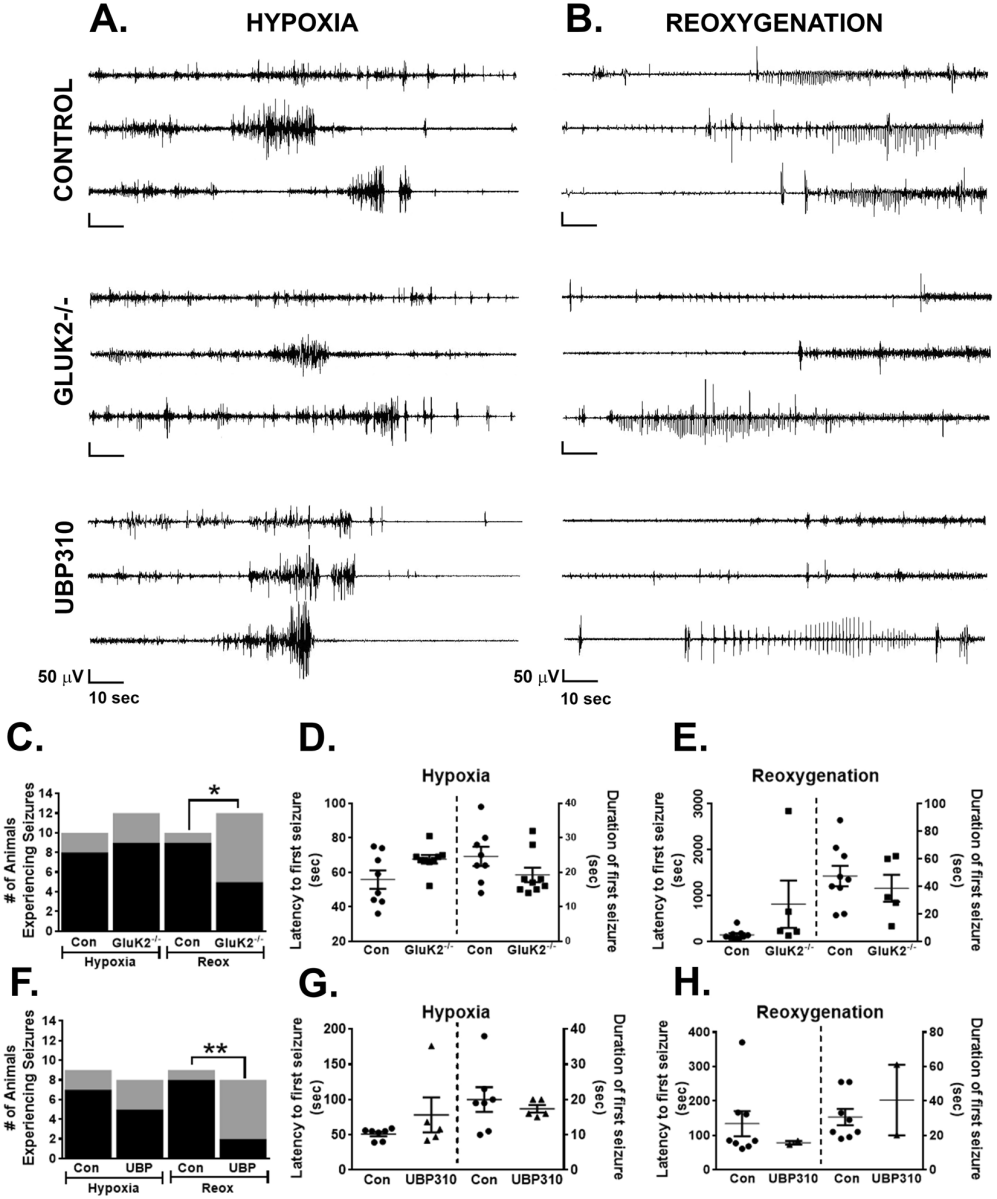
Hypoxia-induced seizures in control, Gluk2^−/−^ and UBP310-treated neonatal mice. (**A**) Representative hippocampal EEG traces during the hypoxic period in control, GluK2^−/−^ and UBP310-treated mice. The initial 2 minutes of hypoxia are shown in 3 representative mice for each group, demonstrating seizures in some animals followed by background suppression in all. Of note seizures during this time period are clinically evident with rhythmic head and forelimb clonic jerks. (**B**) Representative hippocampal EEG traces during the reoxygenation period in control, GluK2^−/−^ and UBP310-treated mice. The initial 2 minutes of reoxygenation are shown in 3 representative mice for each group, demonstrating seizures in some animals. The majority of seizures observed during reoxygenation are electrographic only, emphasizing the need for EEG monitoring in this developmental period. (**C**) Bar graph depicting the occurrence of seizures in control (Con, *n* = 10) and GluK2^−/−^ (*n* = 12) neonatal mice during hypoxia and reoxygenation (*p = 0.027, Chi-square; black = seizure, grey = no seizures). (**D**,**E**) Scatter plots showing seizure latency and seizure duration as mean ± SEM in control and GluK2^−/−^ mice during hypoxia (**D**). and reoxygenation (**E**). There were no statistical differences in seizure latency or duration between the two groups (*p* = 0.054 and 0.138 and *p* = 0.199 and 0.483 for latency and duration of hypoxic and reoxygenation seizures, respectively, *t*-test). (**F**) Bar graph depicting the occurrence of seizures in control (Con, *n* = 9) and UBP310-treated (UBP, *n* = 8) neonatal mice during hypoxia and reoxygenation (**p = 0.0075, Chi-square; black = seizure, grey = no seizures). (**G,H**) Scatter plots showing seizure latency and seizure duration as mean ± SEM in control mice as well as mice pre-treated with UBP310 during hypoxia (**G**) and reoxygenation (**H**). There were no statistical differences in seizure latency or duration between the two groups (*p* = 0.222; 0.559 and *p* = 0.490; 0.465 for latency and duration of hypoxic and reoxygenation seizures, respectively, *t*-test). ● = control (Con), ■ = GluK2^−/−^, ▲ = UBP310-treated, 20 mg/kg.

Previous studies have reported that GluK2^−/−^ mice demonstrate reduced susceptibility to both kainate^39^ and pilocarpine-induced seizures^31^. To determine the role of GluK2-containing KARs in neonatal hypoxia, we utilized an *in vivo* model which reliably produces seizures during both the hypoxic and early reoxygenation phases^40^, and examined whether GluK2^−/−^ mice have fewer seizures than wild-type control mice (Fig. 1A,B). Also, see Supplemental Table S1 for a complete list of seizure data from each experimental animal.

During the hypoxic period, we found that 80% of control mice (*n* = 10) compared to 75% of GluK2^−/−^ mice (*n* = 12, *p* non-significant, Chi-square, Fig. 1C) experienced at least one seizure. There were no differences in seizure latency (55.8 ± 5.3 sec vs. 67.6 ± 2.5 sec, control vs. GluK2^−/−^ mice, respectively, *p* = 0.054, *t*-test) or seizure duration (24.6 ± 2.8 sec vs. 19.2 ± 2.1 sec, control vs. GluK2^−/−^ mice, respectively, *p* = 0.137, *t*-test) between the two groups (Fig. 1D). In contrast, during the reoxygenation period, 90% of control mice, compared to 41.6% of GluK2^−/−^ mice (*p* = 0.027, Chi-square, Fig. 1C) experienced at least one seizure. Again, no differences were observed in seizure latency (142.7 ± 36.6 sec vs. 811.4 ± 515.2 sec, control vs. GluK2^−/−^ mice, respectively, *p* = 0.099, *t*-test) or seizure duration (47.6 ± 7.4 sec vs. 38.6 ± 9.8 sec, control vs. GluK2^−/−^ mice, respectively, *p* = 0.482, *t*-test) between the two groups (Fig. 1E). These findings suggest that GluK2-containing KARs contribute, at least in part, to hypoxia-induced neonatal seizures.

### Neonatal mice pre-treated with UBP310 are less susceptible to seizures during the reoxygenation period following hypoxia

**Figure 2:**
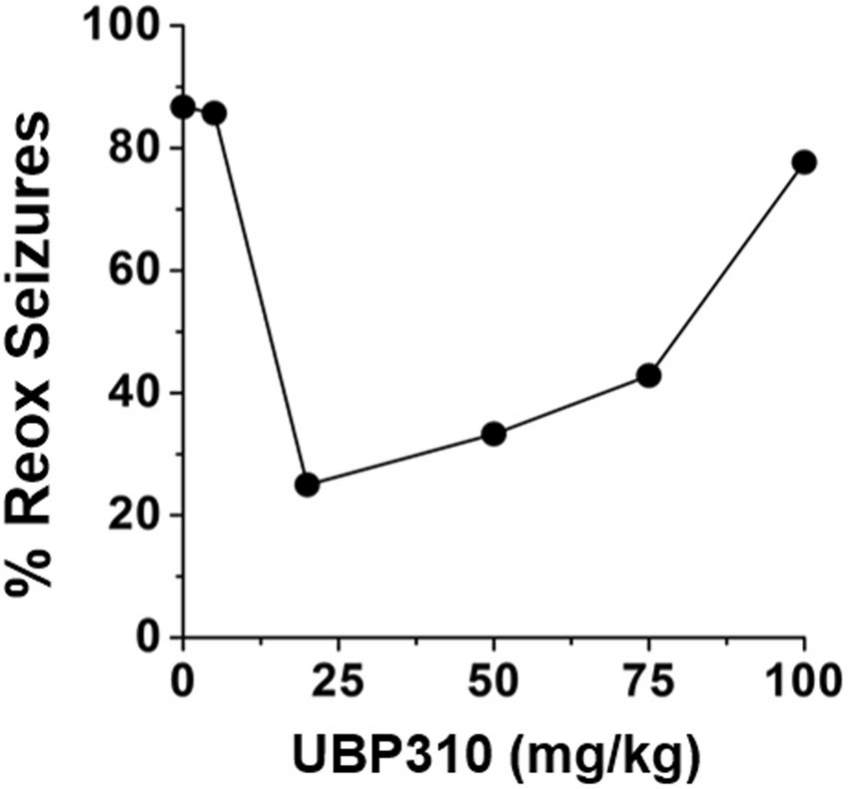
A dose response curve to UBP310 in neonatal P7–9 mice reveal a U-shaped curve. Nearly 90% of mice who did not receive UBP310 experienced a seizure during the reoxygenation period. This result was similar to those mice that received either 5 mg/kg (86%, *n* = 7) or 100 mg/kg (78%, *n* = 10). Following 50 and 75 mg/kg administration, reoxygenation seizures decreased to 33.3% (*n* = 7) and 42.8% (*n* = 7) of mice, respectively. A dose of 20 mg/kg achieved maximal results and reduced seizures by 75% (*n* = 8). Therefore, subsequent experiments utilized 20 mg/kg UBP310. Note, UBP310 was administered subcutaneously 30 min prior to onset of hypoxia.

To confirm and extend our previous findings, we examined whether pharmacological blockade of KARs with UBP310 would impact seizure susceptibility following a hypoxic insult. UBP310 has been reported to be a potent antagonist at heteromeric GluK2/5-containing KARs, as well as GluK1 and GluK3 containing KARs^17,31,41–43^. Additionally, UBP310 has been shown to reduce the number of interictal spikes and seizure frequency in adult epileptic mice^31^. To determine the optimal dose of UBP310 in neonatal mice, a dose-response curve was performed (Fig. 2). We found that a dose of 20 mg/kg achieved maximal results and reduced seizures by 75% (*n* = 8) and therefore, was utilized for all subsequent experiments. Mass spectrometry was used to confirm that UBP310 (20 mg/kg) administered subcutaneously (SC) can penetrate the blood-brain barrier in neonatal mice (Supplemental Table S3). Also see Supplemental Table S2 for a complete list of seizure data from each experimental animal.

In agreement with our previous set of control mice, we observed a significant seizure burden during the hypoxic period, with 77.8% of control animals (*n* = 9) experiencing seizures. Similar to what we observed in GluK2^−/−^ mice, pre-treatment with UBP310 did not decrease seizure occurrence during hypoxia with 62.5% of mice experiencing at least one seizure (*n* = *8, p* non-significant, Chi-square, Fig. 1F). Seizure latency was similar between control (51.0 ± 3.1 sec) and UBP310-treated mice (78.2 ± 24.9 sec, *p* = 0.22, *t*-test), as was seizure duration (20.0 ± 3.5 sec vs. 17.4 ± 1.1 sec in control and UBP310-treated mice, respectively, *p* = 0.56, *t*-test; Fig. 1G). However, during the reoxygenation period, only 25% of mice pre-treated with UBP310 experienced at least one seizure versus 88.9% in control animals (*p* = 0.0075, Chi-square, Fig. 1F). Seizure latency during this period was decreased in UBP310 treated animals (134.0 ± 36.7 sec vs. 78.5 ± 5.5 sec, control vs. UBP310-treated, respectively, *p* = 0.03, *t*-test). The duration of seizures during the reoxygenation period was not different (30.6 ± 4.8 sec vs. 40.5 ± 20.5 sec in control and UBP310-treated mice, respectively, *p* = 0.56, *t*-test) between the two groups (Fig. 1H). Given the significant reduction in seizure incidence during the reoxygenation period in UBP310-treated animals, only 2 seizures were observed limiting the value of the data regarding seizure latency and duration.

### Blockade of KARs does not affect baseline EEG characteristics

**Figure 3:**
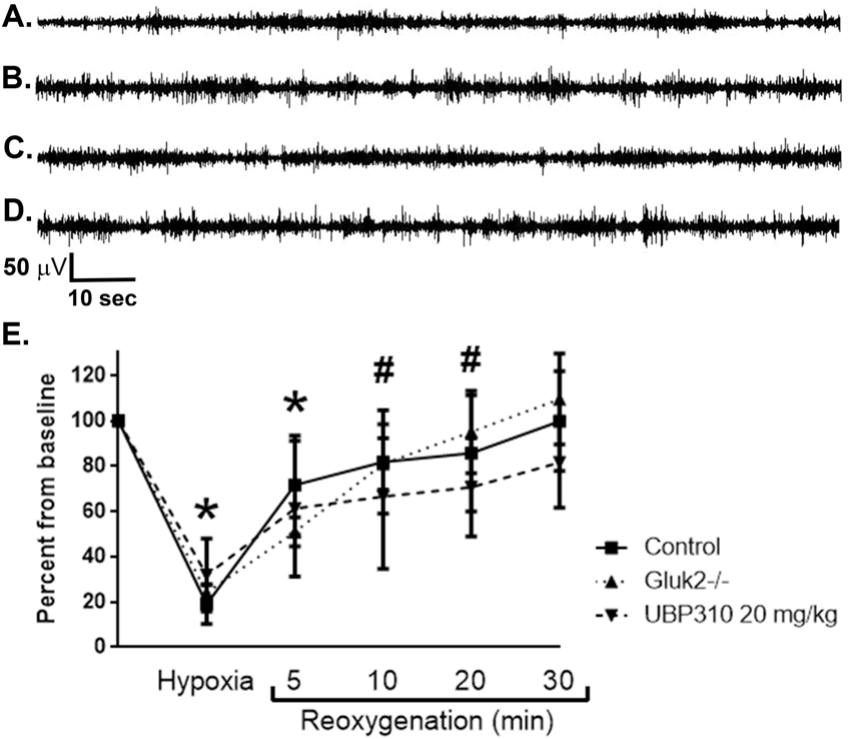
Effects of hypoxia on background EEG characteristics in control, GluK2^−/−^ and UBP310-treated neonatal mice. (**A–D**) Representative hippocampal EEG traces at baseline demonstrating background EEG patterns in control mice (**A**); GluK2^−/−^ mice (**B**); and control pre- and post-UBP310 injection in the same animal (**C** and **D**, respectively). In all groups, background activity consisted of an almost continuous pattern with interspersed sharps and brief periods of low amplitude activity. (**E**) Exposure to 4% hypoxia for 4 min and 30 sec resulted in significant background attenuation in control (*n* = 11), UBP310-treated (*n* = 14) and GluK2^−/−^ mice (*n* = 8; *p < 0.0001 vs. baseline in all groups, ANOVA). A progressive return to baseline was observed over the first 30 min of reoxygenation. Background activity remained significantly suppressed at 5 min in all groups (*p < 0.0001 vs. baseline in all groups, ANOVA) and at 10 and 20 min after start of reoxygenation in GluK2^−/−^ and UBP310-treated mice (^#^*p* < 0.0001 vs. baseline, ANOVA). By 30 min after the start of reoxygenation, background activity was not different than pre-hypoxia. There was no significant difference between groups either with degree of background suppression or timing of return to baseline (*p* = 0.869, ANOVA). For all analyses of the effects of hypoxia on background EEG activity, sections of EEG without artefacts or seizures were selected at predetermined time points as indicated in the method section. Data shown as percent mean ± SEM.

We next sought to determine if the absence of the GluK2 subunit impacted background EEG characteristics at baseline, during hypoxia or reoxygenation. As previously described^40^, we found that baseline EEG activity in neonatal (P7 – P9) control mice consisted of nearly continuous activity with interspersed spikes and brief periods of low amplitude activity. A similar baseline activity pattern was observed in GluK2^−/−^ mice and control mice treated with UBP310 (Fig. 3A–D). Exposure to *in vivo* hypoxia resulted in significant background attenuation in all groups of mice, with progressive improvement in background activity and return to pre-hypoxia baseline activity by 30 minutes after hypoxia onset (Fig. 3E). Please see Supplemental Fig. S1 for representative power spectrum analysis from control, GluK2^−/−^, and UBP310-treated mice. Of note, seizures during the hypoxic period are clinically evident with rhythmic head and forelimb clonic jerks, while the majority of seizures observed during the reoxygenation period are electrographic only, emphasizing the need for EEG monitoring during this early developmental period. These data demonstrate that the presence of the GluK2 subunit does not impact baseline, background EEG characteristics.

Together these results demonstrate that KARs contribute to the seizures observed during the reoxygenation period, but not the hypoxic period, suggesting that different mechanisms underlie seizure generation during these two critical periods. Through both a genetic and pharmacological approach, we have demonstrated for the first time that KARs, likely those containing the GluK2 subunit, mediate, at least in part, hypoxia-induced seizures in the neonatal mouse, specifically during the period of reoxygenation.

### GluK2/3 subunit expression is increased following *in vivo* hypoxia in hippocampal area CA3 in the neonatal mouse

**Figure 4:**
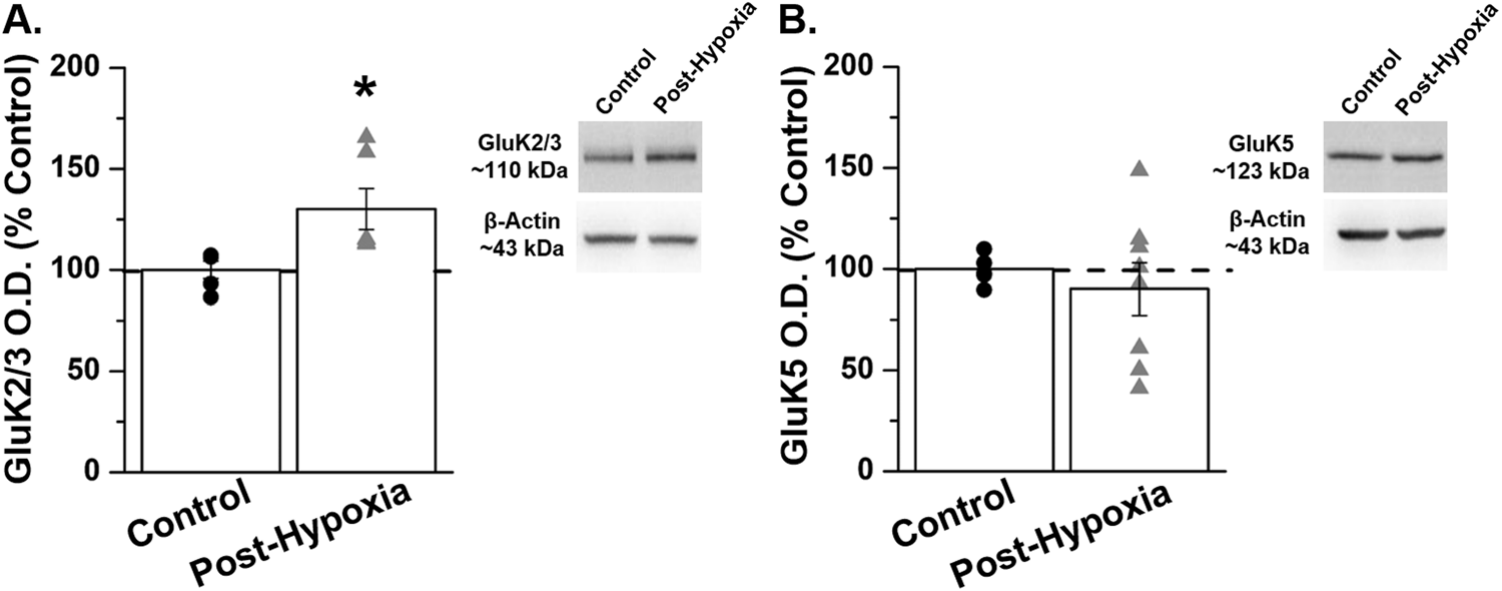
GluK2/3 subunit expression is significantly increased in area CA3 of the neonatal mouse post-hypoxia. (**A**) Expression of GluK2/3 is significantly increased in area CA3 of the neonatal mouse 1-hour post *in vivo* hypoxia. (**B**) GluK5 subunit expression is not altered in area CA3 1-hour post *in vivo* hypoxia in the neonatal mouse. *n* = 5–8 pooled samples (microdissected CA3 regions from two littermate pups, *n* = 1); *p = 0.031, *t-*test. Data shown as percent mean ± SEM. Please see Supplemental Fig. S2 for full-length gel images.

Given that genetic or pharmacological blockade of KARs significantly reduced hypoxia-induced seizures, we sought to determine if KAR subunit expression is altered following *in vivo* hypoxia. The heteromeric GluK2/5 receptor is the most predominant subunit combination in area CA3 and the GluK5 subunit has been reported to be involved in trafficking of KARs^17,18,44^. For these reasons we chose to examine subunit expression of both the GluK2 and GluK5 subunits following *in vivo* hypoxia. We found that one hour following exposure to *in vivo* hypoxia, expression of the GluK2/3 subunit increased to 130.1 ± 10.1% of control, *p* = 0.031, *t*-test), whereas GluK5 subunit expression remained unchanged (90.1 ± 13% of control, *p* = 0.53, *t*-test; Fig. 4A,B; Supplemental Fig. S2). These data demonstrate that expression of GluK2, one of the most predominant KAR subunits is significantly increased in area CA3 of the neonatal mouse hippocampus following hypoxia. Based on our previous EEG recordings in which knockout of the GluK2 subunit reduced seizures only during the reoxygenation period, we suggest that the observed increase in GluK2 subunit expression likely occurs during the reoxygenation period following a hypoxic insult.

### Treatment with UBP310 prevents the increase in mEPSC frequency following exposure to *in vivo* hypoxia in the neonatal mouse

**Figure 5:**
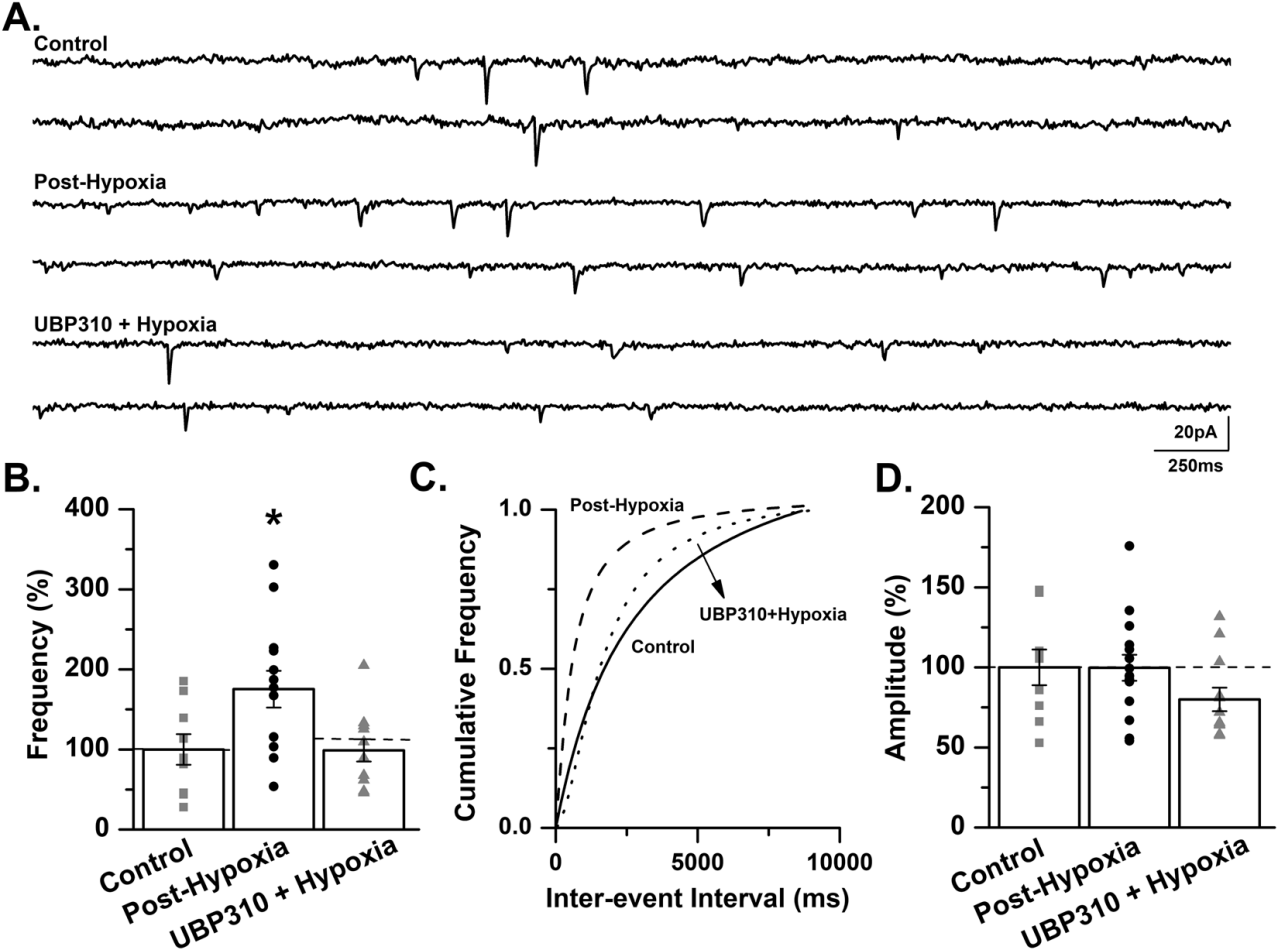
Treatment with UBP310 prevents the increase in mEPSC frequency following exposure to *in vivo* hypoxia in the neonatal mouse. (**A**) Representative traces from a control C57BL/6 mouse (Control), a mouse 1-hour post *in vivo* hypoxia (Post-Hypoxia) and a mouse exposed to *in vivo* hypoxia following treatment with 20 mg/kg UBP310 (UBP310 + Hypoxia). Traces demonstrate an increase in mEPSC frequency post-hypoxia with similar frequencies between control and UBP310-treated animals. (**B**) mEPSC frequency is significantly increased in CA3 pyramidal cells 1-hour post-hypoxia. Treatment with UBP310 prior to hypoxia prevents the increase in mEPSC frequency (control: 0.5 ± 0.1 Hz; post-hypoxia: 0.88 ± 0.12 Hz; UBP310 + hypoxia: 0.5 ± 0.07 Hz). *p < 0.05, K-W ANOVA. Data presented as percent mean ± SEM. (**C**) A representative inter-event cumulative frequency histogram demonstrating the significant increase in mEPSC frequency 1-hour post-hypoxia in CA3 pyramidal cells of the neonatal mouse. Control, solid line; Post-Hypoxia, dashed line; UBP310 + Hypoxia, dotted line. (**D**) No change in mEPSC amplitude was observed between groups (control: 14.2 ± 1.58 pA; post-hypoxia: 14.16 ± 1.15 pA; UBP310 + Hypoxia: 11.36 ± 1.04 pA). Control: *n* = 9 cells, 7 animals; Post-Hypoxia: *n* = 13 cells, 11 animals; UBP310 + Hypoxia: *n* = 12 cells, 9 animals. Data presented as percent mean ± SEM.

Based on our observation that the GluK2 subunit expression significantly increased following hypoxia we sought to determine if this increase contributed to changes in excitatory neurotransmission in CA3 pyramidal cells. One hour following exposure to a single hypoxic insult, mEPSCs were recorded from CA3 pyramidal cells. We observed a significant increase in mEPSC frequency (175.4 ± 22.9% of control, *p* < 0.05, ANOVA; Fig. 5A–C) in slices from mice exposed to hypoxia, with no changes in mEPSC amplitude (99.7 ± 8.1% of control, *p* > 0.05, ANOVA; Fig. 5D). This increase in excitatory neurotransmission may represent one of the underlying mechanisms of hypoxia-induced seizures in the neonatal mouse.

Our data show that mice treated with UBP310 experienced significantly fewer seizures during the reoxygenation period (Fig. 1). Given the increase in mEPSC frequency following *in vivo* hypoxia, we sought to determine if treatment with UBP310 prior to hypoxia altered mEPSC frequency. Mice were pretreated with UBP310 (20 mg/kg, SC) 30 minutes prior to exposure to hypoxia. In this group of animals, mEPSC frequency was similar to levels observed in control mice (98.9 ± 13.9% of control, *p* < 0.05, ANOVA; Fig. 5A–C). No change in mEPSC amplitude was observed (80.0 ± 7.3% of control, *p* > 0.05, ANOVA; Fig. 5D). These experiments demonstrate that KARs contribute, at least in part, to the observed increase in excitatory neurotransmission following a single hypoxic insult in the neonatal mouse. In conjunction with our anatomical data, we suggest that a hypoxic insult results in an increase in GluK2-containing KARs, which likely contributes to the observed increase in excitatory neurotransmission and may lead to increased seizure susceptibility following hypoxia in the neonatal mouse.

### OGD increases excitatory synaptic transmission in CA3 pyramidal cells of the neonatal mouse

**Figure 6:**
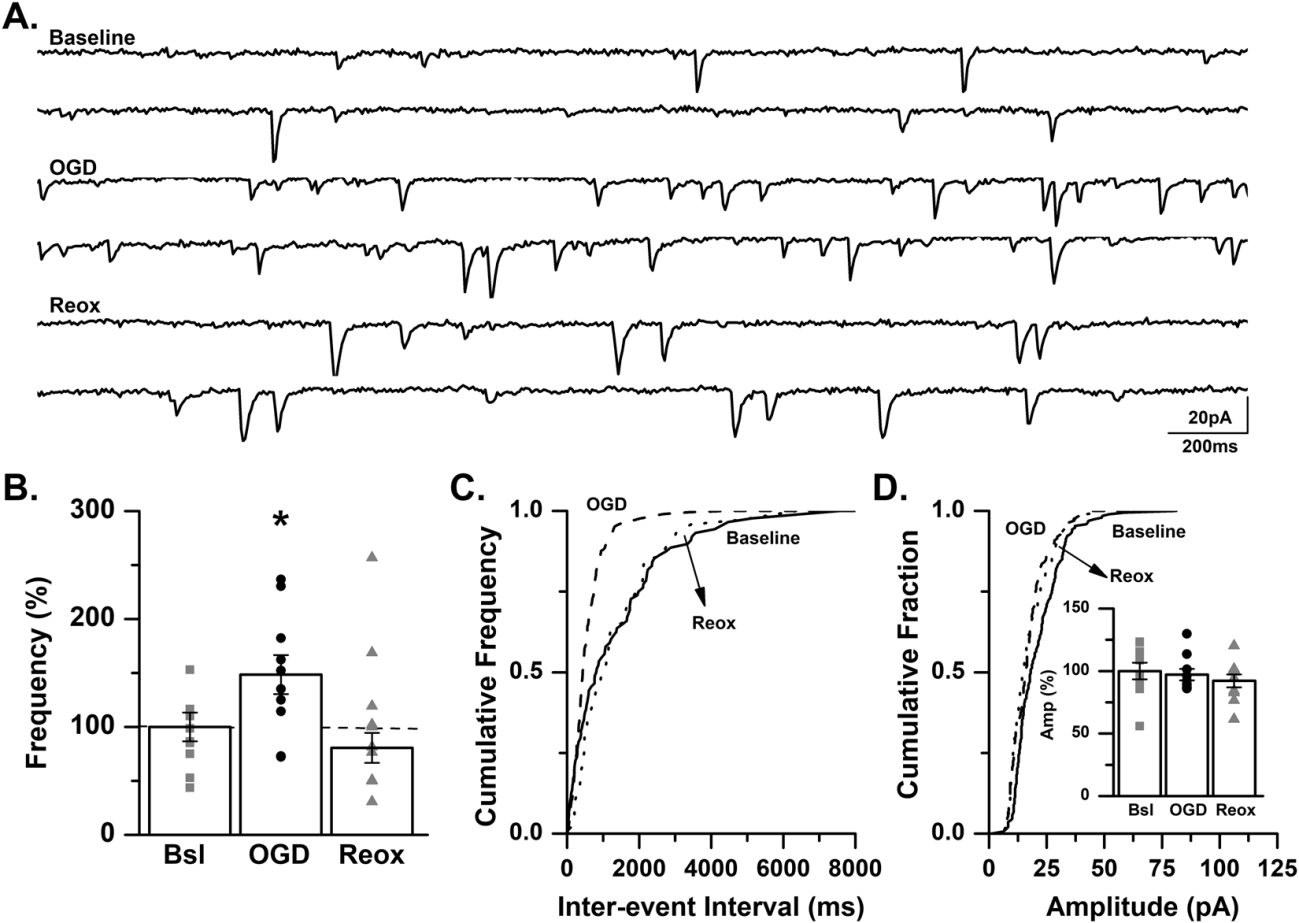
sEPSC frequency is significantly increased in CA3 pyramidal cells during OGD. (**A**) Representative traces from a control C57BL/6 mouse recorded at baseline, during OGD and during reoxygenation (reox) demonstrating an increase in spontaneous EPSC frequency during OGD. (**B**) Averaged data demonstrating that sEPSC frequency is significantly increased during OGD and returns to baseline levels during reox as a percent of baseline (*n* = 10 cells, 6 animals; baseline, Bsl: 1.4 ± 0.18 Hz; OGD: 2.08 ± 0.25 Hz; reox: 1.13 ± 0.19 Hz). *p < 0.05, K-W ANOVA. Data presented as percent mean ± SEM. (**C**) Representative inter-event cumulative frequency histogram demonstrating a reduction in the inter-event interval during OGD. (**D**) Representative amplitude cumulative histogram demonstrating no change in mEPSC amplitude during OGD. Inset showing averaged data for sEPSC amplitude at baseline, during OGD and during reox. (baseline, Bsl: 17.06 ± 1.14 pA; OGD: 16.57 ± 0.77 pA; reox: 15.72 ± 0.88 pA). Data presented as percent mean ± SEM. Baseline, solid line; OGD, dashed line; reox, dotted line.

We have previously demonstrated that oxygen-glucose deprivation (OGD), an *in vitro* hippocampal slice model of hypoxic-ischemia, increases excitatory neurotransmission in CA1 pyramidal cells of the neonatal mouse^45^. Consistent with this previous study, we report that spontaneous EPSC (sEPSC) frequency is significantly increased in CA3 pyramidal cells of neonatal (P7–9) mice during OGD (148.4 ± 18.0% of baseline, *p* < 0.05, ANOVA; Fig. 6A–C). During the period of reoxygenation sEPSC frequency was not significantly different than baseline levels (80.6 ± 13.9% of baseline, *p* > 0.05, ANOVA; Fig. 6A–C). No changes in sEPSC amplitude were observed (OGD: 97.1 ± 0.5% of baseline; reoxygenation: 92.2 ± 0.5% of baseline, *p* > 0.05, ANOVA; Fig. 6D).

**Figure 7:**
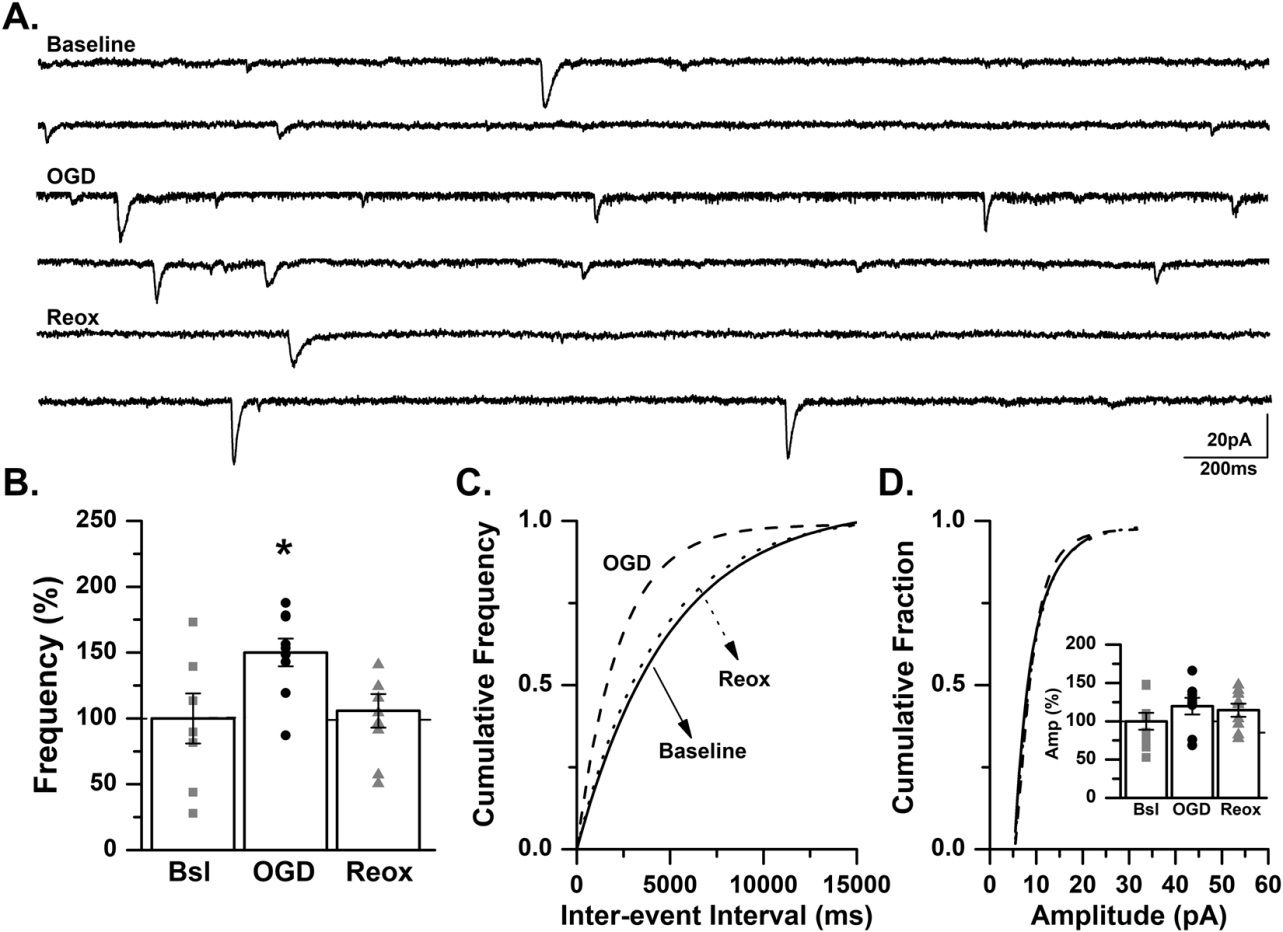
mEPSC frequency is significantly increased in CA3 pyramidal cells during OGD. (**A**) Representative traces from a control C57BL/6 mouse recorded at baseline, during OGD and during reoxygenation (reox) demonstrating an increase in mEPSC frequency during OGD. (**B**) Averaged data demonstrating that mEPSC frequency is significantly increased during OGD and returns to baseline levels during reox as a percent of baseline (*n* = 9 cells, 7 animals; baseline, Bsl: 0.5 ± 0.1 Hz; OGD: 0.72 ± 0.14 Hz; reox: 0.45 ± 0.06 Hz). *p < 0.05, K-W ANOVA. Data presented as percent mean ± SEM. (**C**) Representative inter-event cumulative frequency histogram demonstrating a reduction in the inter-event interval during OGD. (**D**) Representative amplitude cumulative histogram demonstrating no change in mEPSC amplitude during OGD (baseline, Bsl: 14.2 ± 1.58 pA; OGD: 16.14 ± 1.54 pA; reox: 15.8 ± 1.91 pA). Inset showing averaged data for mEPSC amplitude at baseline, during OGD and during reox. Data presented as percent mean ± SEM. Baseline, solid line; OGD, dashed line; reox, dotted line.

We next included tetrodotoxin (TTX) a Na^+^ channel blocker in our recording solution to examine action-potential independent release. Similar to results observed with sEPSCs, the frequency of mEPSCs in CA3 pyramidal cells increased significantly during OGD (150.1 ± 10.5% of baseline, *p* < 0.05, ANOVA) followed by a return to baseline frequency during reoxygenation (105.8 ± 12.7% of baseline, *p* > 0.05, ANOVA; Fig. 7A–C). No significant difference in mEPSC amplitude was observed during OGD or during the reoxygenation period (OGD: 119.7 ± 10.1% of control; reoxygenation: 114.5 ± 8.5% of control, *p* > 0.05, ANOVA; Fig. 7D). These data suggest that presynaptic mechanisms contribute, at least in part, to the increased frequency of mEPSCs observed in CA3 hippocampal neurons from neonatal mice. In conjunction with our previous report in CA1 pyramidal cells^45^, these experiments strongly suggest that OGD results in increased hippocampal neuronal excitability and may contribute to the generation of epileptiform activity.

### The increase in mEPSC frequency during OGD is absent in a neonatal GluK2^−/−^ mouse

**Figure 8:**
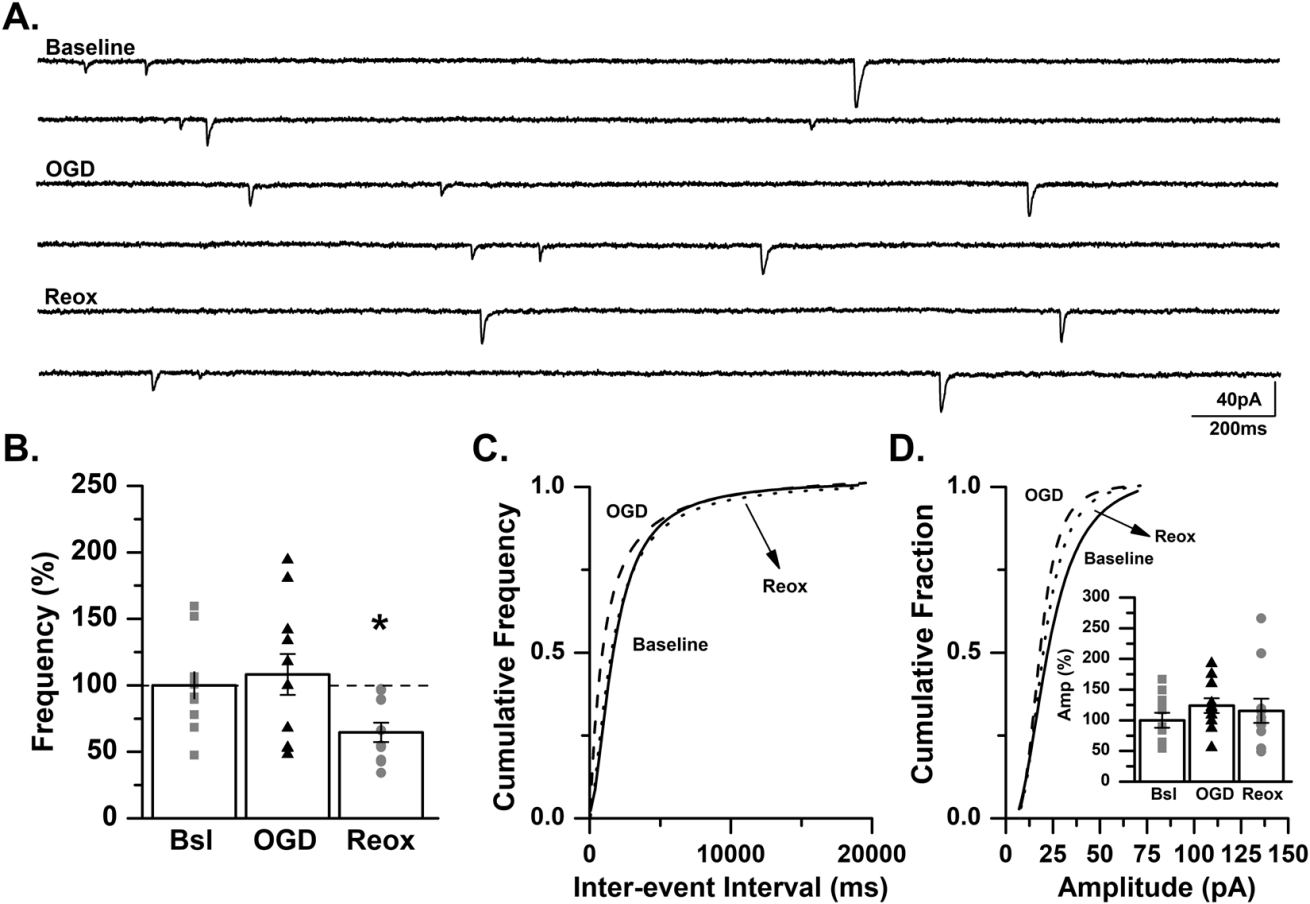
Increase in mEPSC frequency during OGD is absent in a GluK2^−/−^ mouse. (**A**) Representative traces from a GluK2^−/−^ mouse recorded at baseline, during OGD and during reoxygenation (reox) demonstrating that mEPSC frequency is not changed during OGD. (**B**) Averaged data demonstrating that mEPSC frequency is not altered during OGD but is significantly reduced during reox as a percent of baseline (*n* = 11 cells, 9 animals; baseline, Bsl: 0.53 ± 0.05 Hz; OGD: 0.58 ± 0.1 Hz; reox: 0.34 ± 0.05 Hz). *p < 0.05, K-W ANOVA. Data presented as percent mean ± SEM. (**C**) Representative inter-event cumulative frequency histogram demonstrating no change in the mEPSC frequency in a GluK2^−/−^ mouse. (**D**) Representative amplitude cumulative histogram demonstrating no change in mEPSC amplitude during OGD. Inset showing averaged data for mEPSC amplitude at baseline, during OGD and during reox, (baseline, Bsl: 15 ± 1.82 pA; OGD: 17.41 ± 1.8 pA; reox: 15.71 ± 2.2 pA). Data presented as percent mean ± SEM. Baseline, solid line; OGD, dashed line; reox, dotted line.

To further investigate the role of KARs in response to OGD further, mEPSCs were recorded as previously described in hippocampal slices obtained from GluK2^−/−^ mice. In these recordings, mEPSC frequency was not altered during OGD (108.2 ± 15.4% of baseline), but was significantly reduced during the reoxygenation phase (64.6 ± 7.3% of baseline, *p* < 0.05, ANOVA; Fig. 8A–C). We did not observe a change in mEPSC amplitude (OGD: 124.0 ± 11.9% of baseline; reoxygenation: 115.4 ± 19.7% of baseline, *p* > 0.05, ANOVA; Fig. 8D). These data from an *in vitro* hippocampal slice model of hypoxic-ischemia, suggest that GluK2-containing KARs contribute to periods of both OGD and reoxygenation as observed by changes in mEPSC frequency in CA3 pyramidal cells of the neonatal mouse.

### GluK2-containing KARs contribute to increased mEPSC frequency following OGD in the neonatal mouse

**Figure 9:**
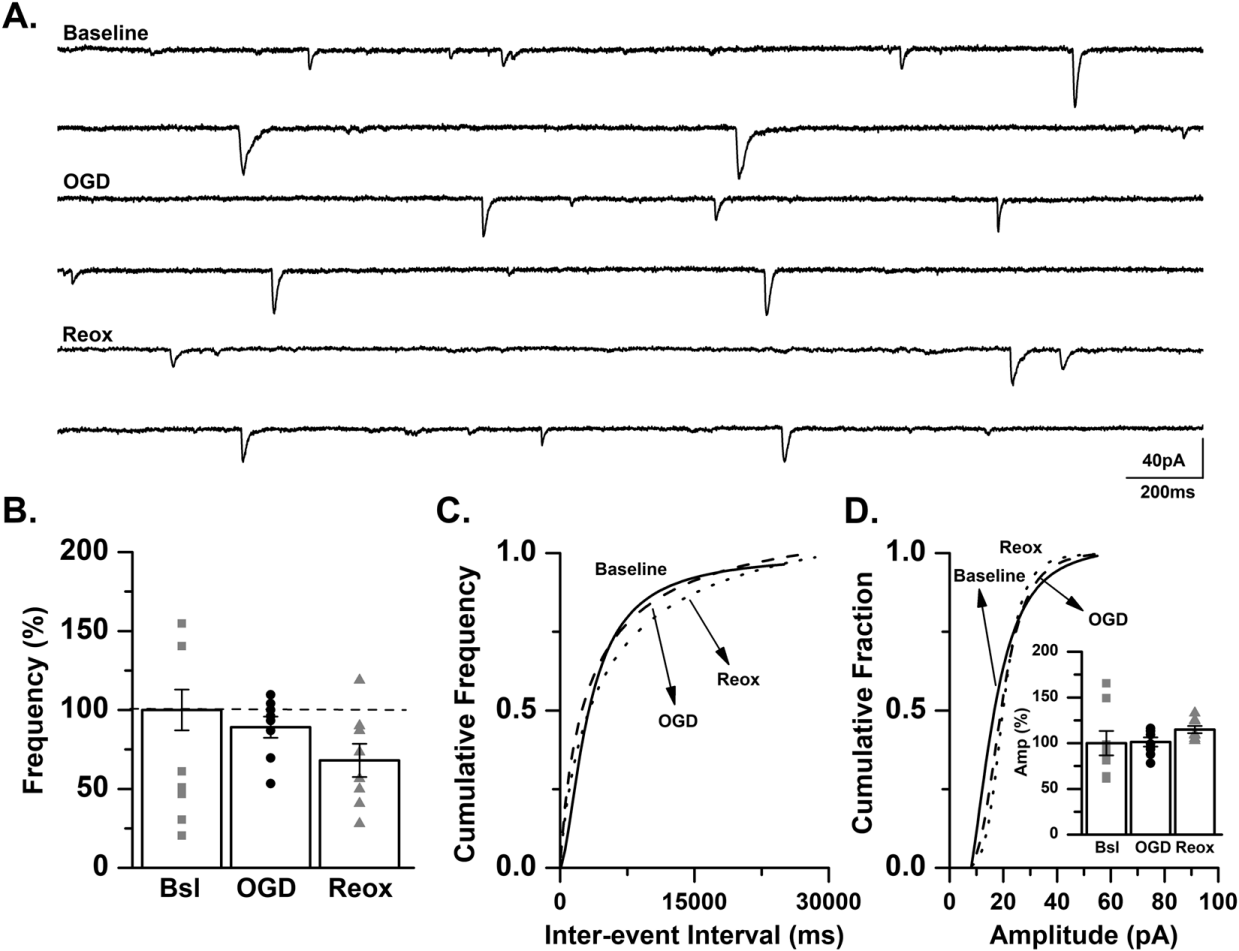
UBP310 prevents the increase in mEPSC frequency during OGD. (**A**) Representative traces from a control C57BL/6 mouse recorded at baseline, during OGD and during reoxygenation (reox) demonstrating no change in mEPSC frequency during OGD. (**B**) Averaged data for mEPSC frequency during OGD and reox as a percent of baseline (*n* = 8 cells, 6 animals; baseline, Bsl: 0.49 ± 0.16 Hz; OGD: 0.46 ± 0.18 Hz; reox: 0.37 ± 0.15 Hz). (**C**) Representative inter-event cumulative frequency histogram showing no change in the inter-event intervals during OGD and reox. (**D**) Representative amplitude cumulative histogram demonstrating no change in mEPSC amplitude during OGD. Inset showing averaged data for mEPSC amplitude during OGD and reox as a percent of baseline (baseline, Bsl: 13.0 ± 1.74 pA; OGD: 13.19 ± 1.9 pA; reox: 15.03 ± 2.21 pA). Data presented as percent mean ± SEM. Baseline, solid line; OGD, dashed line; reox, dotted line.

To confirm this finding pharmacologically, mEPSCs were recorded from hippocampal slices obtained from control mice and treated with UBP310. Blockade of KARs with UBP310 (5 µM) prevented the anticipated increase in mEPSC frequency during OGD (89.1 ± 6.7% of baseline, *p* > 0.05, ANOVA). No change in mEPSC frequency was observed during the reoxygenation period (68.0 ± 10.6% of baseline, *p* > 0.05, ANOVA; Fig. 9A–C). No change in mEPSC amplitude was observed during OGD or reoxygenation with the inclusion of UBP310 to the drug cocktail (OGD: 101.3 ± 4.9% of baseline; reoxygenation: 114.8 ± 3.9% of baseline, *p* > 0.05, ANOVA; Fig. 9D). Given that the results obtained in the presence of UBP310 are similar to those observed in a GluK2^−/−^, we conclude that KARs contribute to changes in excitatory neurotransmission in area CA3 of the neonatal mouse hippocampus, which likely contributes to the generation of hypoxia-associated seizures.

## Discussion

The present study examined the role of KARs in mediating seizures and in modulating excitatory neurotransmission following a hypoxic insult the neonatal mouse. We report that in an *in vivo* hypoxia model, neonatal mice lacking the GluK2 subunit of the KAR were significantly less susceptible to seizures, specifically during the reoxygenation period. In addition, pre-treatment with UBP310, a KAR antagonist, produced a similar reduction in seizure susceptibility, further supporting a role of KARs in regulating hypoxia-induced neonatal seizures. Importantly, blockade of KARs resulted in an attenuation of the hypoxia-induced increase in excitatory neurotransmission observed in CA3 pyramidal cells of the neonatal mouse hippocampus.

In a separate *in vitro* hippocampal slice model of hypoxic-ischemia, composed of a period of oxygen-glucose deprivation (OGD) and subsequent reoxygenation, we observed a significant increase in mEPSC frequency during the period of OGD with a return to baseline levels during reoxygenation. This increase in frequency during OGD was absent in recordings from slices from GluK2^−/−^ mice or control mice treated with UBP310 prior to hypoxia. Together, these data demonstrate for the first time that KARs contribute, at least in part, to the seizures associated with hypoxia through regulation of excitatory neurotransmission in area CA3 of the neonatal hippocampus.

Through Western blot analysis, we found that GluK2/3 subunit expression was significantly increased in the hippocampal CA3 region of neonatal mice 1 – hour following *in vivo* hypoxia. Our observation of rapid changes in protein translation is in agreement with previous studies^46,47^, and demonstrates that exposure to a single hypoxic insult significantly alters KAR subunit expression. Our data, demonstrating that GluK2/3 subunit expression is significantly increased post *in vivo* hypoxia and that blockade of the GluK2 subunit reduces susceptibility to seizures during the reoxygenation period, is in agreement with previous studies in which epileptic mice lacking the GluK2 subunit were reported to display a reduction in the number of seizures per day^31^ and were less susceptible to kainate-induced seizures^39^. Future studies will examine more long-term changes in KAR subunit expression and will determine if our observation of rapid changes in GluK2/3 subunit expression post *in vivo* hypoxia are enduring.

The GluK2^−/−^ mouse, first published by Mulle, *et al*.^39^, has proven to be an immensely valuable tool for advancing our understanding of the role of KARs in regulating synaptic neurotransmission in both healthy and diseased brains^26,32,34^. In agreement with previous studies^48–51^, we observed a significant decrease in expression of the GluK5 subunit in GluK2^−/−^ mice (56.1 ± 2.5% of control, *p* = 0.0005; Supplemental Fig. S3). This decrease substantiates previous conclusions that the GluK2 subunit partially mediates GluK5 expression and is critical for subunit stabilization in CA3 pyramidal cells^48,52^. Therefore KARs in area CA3 of neonatal GluK2^−/−^ mice were likely comprised of homomeric GluK2 or heteromeric GluK2/3 KARs, a conclusion that is supported by our studies utilizing the willardine-derivative UBP310. Although initially developed as a GluK1 antagonist^41^ UBP310 has since been reported to inhibit heteromeric GluK2/5 receptors as well as GluK3-containing KARs^17,31,42^ It should be noted that expression levels of the GluK1 subunit are elevated in the immature brain^53^, and thus inhibition by UBP310 may in part be due to its antagonistic action at GluK1-containing KARs.

Similar to the GluK5 subunit, expression of the KAR auxiliary proteins Neto1 and Neto2, which regulate synaptic localization of KARs as well as the gating and affinity of both homomeric and heteromeric KARs^51,54–57^, are also significantly decreased in GluK2^−/−^ mice^51,58^. Future experiments examining potential alterations in the expression of Neto1 and Neto2 may provide additional insight into changes in KAR subunit expression and subunit composition following exposure to neonatal hypoxia. Furthermore, as KARs composed of GluK1–3 subunits have been identified in interneurons^15,59–61^ it is possible that hippocampal interneuron activity and inhibitory neurotransmission may be impacted following a hypoxic insult. These possibilities will be explored in future experiments targeted at further examining the role of hippocampal KARs during hypoxic-ischemic events in the neonatal brain.

To confirm our findings of reduced seizure susceptibility in GluK2^−/−^ mice, we utilized the KAR antagonist UBP310. A previous study reported that intraperitoneal injection (IP) of UBP310 (60 mg/kg) significantly reduced the frequency of interictal spikes and the number of seizures per day in adult epileptic mice^31^. We found that in neonatal mice, subcutaneous injection of UBP310 was more reproducible than IP injection due to a significant loss of volume during IP administration. To confirm that subcutaneous injection of UBP310 was effective in decreasing hypoxia-induced seizures, and to determine the maximally effective dose, we performed a dose-response curve (Fig. 2), and determined 20 mg/kg as the appropriate dose. Of interest, we found treatment with UBP310 resulted in an inverted U – shaped dose response curve, with both low (5 mg/kg) and high (100 mg/kg) doses of UBP310 having minimal impact on hypoxia-induced seizures. While the pharmacological mechanism(s) underlying this occurrence remain poorly understood^62,63^, we speculate that low doses of UBP310 do not sufficiently block KARs, whereas higher does may generate off-target effects. Additionally, we employed mass spectrometry to confirm that a dose of 20 mg/kg of UBP310 sufficiently crossed the blood-brain barrier in neonatal mice. This data, in conjunction with our utilization of GluK2^−/−^ mice, confirm a role of GluK2-containing KARs in the generation of seizures associated with a hypoxic event in neonatal mice.

We observed a significant increase in mEPSC frequency from CA3 pyramidal cells in slices obtained from mice 1-hour post hypoxia, which was subsequently prevented in animals pretreated with UBP310 (Fig. 5). We suggest that the increase in GluK2/3 subunit expression post-hypoxia contributes to the observed increase in mESPC frequency. Therefore, treatment with UBP310 prior to hypoxia either prevents the increase in GluK2/3 expression or acts by inhibiting these newly expressed receptors, thus preventing the increase in mEPSC frequency post-hypoxia. Additional experiments will be required to directly determine the mechanism(s) by which UBP310 prevents the increase in excitatory neurotransmission post hypoxia.

In the *in vivo* hypoxia model, we found that both genetic ablation of the GluK2 subunit, or blockade of KARs with UBP310, significantly reduced the number of animals experiencing seizures during the reoxygenation period, but not the hypoxic period. Because this model does not allow for investigating the effects of hypoxia and reoxygenation on excitatory synaptic transmission independently, we also utilized an *in vitro* OGD model to begin to address these questions. In slices obtained from control mice, we observed an increase in excitatory neurotransmission during the period of OGD with subsequent return to baseline during reoxygenation. In contrast, in slices obtained from GluK2^−/−^ mice or UBP310-treated control mice, mEPSC frequency was significantly decreased or trending towards a reduction, respectively, during the reoxygenation period. These results highlight the important differences between the hypoxic and reoxygenation periods, and suggest that distinct physiological and pharmacological mechanisms underlie the changes observed in excitatory synaptic transmission during these two periods. Nonetheless, these data continue to provide support of a role of KARs in the pathophysiology of seizures associated with a single hypoxic insult in neonatal mice, as blockade of KARs significantly impacted both periods of the hypoxic insult.

Previously, we reported that both pre- and post-synaptic mechanisms contributed to the effects of *in-vitro* OGD in CA1 pyramidal cells from neonatal mice, as both changes in mEPSC frequency and decay kinetics, but not amplitude were observed^45^. In contrast, in the current study we observed a significant decrease in mEPSC frequency, while no change in the decay kinetics (Supplemental Fig. 4) or amplitude of the mEPSC was observed under any conditions examined. Based on these findings, we suggest that presynaptic KARs localized on the mossy fiber terminal and/or the associational/commissural (A/C) pathway mediate the observed increase in excitatory neurotransmission following a hypoxic insult in the neonatal mouse. While future studies are necessary to explicitly determine potential changes in both pre- and post-synaptic KARs, our data is in agreement with other studies highlighting the role of presynaptic GluK2 and GluK3-containing KARs in regulating excitatory neurotransmission^23,64,65^. Data from these two studies demonstrate that different mechanisms, including different receptor populations likely contribute to the differential changes in excitatory synaptic transmission during observed in in areas CA1 and CA3 of the neonatal mouse during hypoxia and reoxygenation.

Postsynaptic KARs mediate a small excitatory current, approximately 8–10% of the peak AMPA current, with significantly slower deactivation kinetics^19,20,66,67^. While some studies have investigated quantal activation of the EPSC_KA_^68,69^ the majority of studies examining postsynaptic KARs utilize stimulation paradigms in order to capitalize on the slow decay kinetics and allow for the generation of larger KAR-mediated currents^17,19,20,70^. In the current study we did not observe the anticipated shift in mEPSC decay kinetics between control and GluK2^−/−^ mice or following administration of the KAR antagonist UBP310 (Supplemental Fig. S4). Together we concluded that we cannot speak directly to potential changes in postsynaptic decay kinetics of the KAR-mediated EPSC. Additionally, this finding suggests AMPA and NMDA-mediated currents may be contributing to the observed increased mEPSC frequency. However, as this change in frequency is blocked by a KAR antagonist and absent in the GluK2^−/−^ mouse, we have concluded that increased excitatory neurotransmission associated with *in vitro* hypoxic-ischemia or a single *in vivo* hypoxic insult is predominately mediated by KARs. Additional studies will be required to definitively determine the potential role of postsynaptic KARs in neonatal seizures.

In summary, we have shown that KARs, likely those containing the GluK2 subunit, contribute to seizures associated with a hypoxic insult through alterations in excitatory neurotransmission in CA3 pyramidal cells of the neonatal mouse. Future studies utilizing high resolution advanced microscopy are underway to identify the specific time course of neuronal activity following exposure to *in vivo* hypoxia. This data would provide powerful clinical information regarding the time-frame of therapeutic intervention for neonates exposed to a hypoxic-insult. We are continuing our efforts to elucidate the role of KARs in mediating the effects of hypoxia-induced seizures in the neonatal brain with the goal of these studies leading to the development of novel therapeutic agents for treatment of these seizures in neonates.

## Methods

### Animals

Animal care and use procedures were carried out in accordance with protocols written under the guidelines of the National Institutes of Health Guide for the Care and Use of Laboratory Animals and approved by the Institutional Animal Care and use Committee at the University of Virginia. C57BL/6 mice were purchased from Charles River Laboratories (Wilmington, MA, USA). Dr. Anis Contractor provided KAR knockout mice (GluK2^−/−^) generated from a C57BL/6 background. The colony was maintained at the University of Virginia. Male and female neonatal mouse pups, postnatal day 7–9 were utilized in the following studies. All animals were housed in a temperature controlled environment with *ad libitum* access to food and water.

### *In vivo* hypoxia protocol

*In vivo* hypoxia was performed in postnatal day (P) 7–9 mice as previously detailed with some modifications^40^. Briefly, pups were placed in a heated, custom-made Plexiglas chamber with stable temperature of 30.5 ± 0.2 °C throughout hypoxia and reoxygenation. The heating pad was used throughout hypoxia and recovery periods to minimize any changes in body temperature resulting from the hypoxic insult. Body temperature measurements performed in a separate set of pups demonstrated stable body temperatures of 36.3 ± 0.04 °C (*n* = 4). Hypoxia was produced by flushing the chamber with N_2_ and compressed air to rapidly reach a fraction of inspired oxygen (FiO_2_) of 4% using an Oxidial to control gas flow (Starr Life Sciences Corp). Target FiO_2_ was maintained for 4 min and 30 sec and continuously monitored via an oxygen sensor (Oxygen logger sensor NUL-205). Reoxygenation was obtained by opening and fanning the chamber with room air resulting in rapid return to 21% FiO_2_^40^. A separate group of mice received subcutaneous injections of UBP310 (20 mg/kg) dissolved in DMSO, 30 minutes prior to exposure to the *in vivo* hypoxia protocol outlined above.

### Surgery and electroencephalogram monitoring

Electroencephalogram (EEG) monitoring was performed as previously described^40^. Briefly, neonatal mice were anesthetized with isoflurane (5% induction, 1.5–2% maintenance) via a specially adapted nose cone and surgery was performed in a clean, ventilated hood with sterile instruments and fields. Subcutaneous bupivacaine was used as needed. Monopolar electrodes (2 in dorsal hippocampus, 2 in cortex and 1 ground) were implanted in anesthetized P7–9 mice using the following coordinates: anterior-posterior: −4.5; medial lateral: ±2 and depth from touch points. After recovery, the mice were connected to a unity gain impedance matching head stage (TLC2274 Quad Low-Noise Rail-To-Rail Operational Amplifier, Texas Instruments, Dallas, TX). The output signal was amplified using a Grass amplifier (Model 1, Natus Neurology Incorporated – Grass Products, Warwick, RI), digitized and recorded for later review using a Stellate Harmonie system (Natus Medical Incorporated, San Carlos, CA). All EEGs were independently reviewed by authors SZ and PW. Electrographic seizures were defined as the appearance of high frequency (>2 Hz) rhythmic sharp wave discharges with an amplitude at least 3 times that of baseline and lasting greater than 10 sec with clear evolution^71–73^. EEG background changes with hypoxia and reoxygenation were quantified as previously described^40^ by calculating the mean and standard deviation of the EEG voltage from EEG samples of 10 sec durations. Sample traces were selected at 3 random time points during baseline recording (including pre- and post-UBP310 injection if applicable), during mid- and end of hypoxia as well as at 5, 10, 20 and 30 min after onset of reoxygenation. If an artifact or a seizure prevented the use of the EEG trace at the pre-prescribed time points, measurements were performed using the artifact-free trace immediately following. All results were normalized to baseline with each animal serving as its own control. To determine differences in seizure latency and duration two separate control groups were utilized for each experimental group (GluK2^−/−^ and UBP310) from animals exposed to hypoxia during the same time frame.

### Brain slice electrophysiology

Coronal hippocampal brain slices were prepared from isoflurane-anesthetized neonatal (P7–9) mice. 300 ${\mu }$m thick slices were sectioned using a vibratome (Leica VT 1200 S, Nussloch, Germany) in ice cold (4 °C), oxygenated (95% O_2_/5% CO_2_) sucrose-based artificial cerebrospinal fluid (aCSF) containing (in mM): 120 sucrose, 2 KCl, 1.1 KH_2_PO_4_, 56 NaCl, 25 NaHCO_3_, 10 d-glucose, 6 MgSO_4_, 0.5 CaCl_2_. Slices were then incubated in oxygenated (95% O_2_/5% CO_2_) aCSF containing (in mM): 126 NaCl, 2 KCl, 1.1 KH_2_PO_4_, 26 NaHCO_3_, 10 d-glucose, 2 MgSO_4_, 1.5 CaCl_2_ (pH 7.4, 305–310 mOsm) for 15–20 min at 37 °C and an additional 20–30 min at room-temperature prior to recordings. A subset of electrophysiological recordings were obtained from mice following exposure to *in vivo* hypoxia. These mice were sacrificed one – hour following the completion of the *in vivo* hypoxia protocol and coronal brain slices were prepared as described. All electrophysiological recordings took place within 1–5 hours following preparation of slices.

For whole-cell patch-clamp recordings, slices were placed in a submerged chamber perfused with warm, oxygenated (30 °C; 95% O_2_/5% CO_2_) standard aCSF containing (in mM): 126 NaCl, 2 KCl, 1.1 KH_2_PO_4_, 26 NaHCO_3_, 10 d-glucose, 2 MgSO_4_, 1.5 CaCl_2_ (pH 7.4, 305–310 mOsm). CA3 pyramidal neurons were visually identified using a Nikon Eclipse E600FN microscope equipped with a 40X water-immersion objective and a high-performance CCD camera. Currents were recorded using an Axopatch 200B amplifier (Axon Instruments, Union City, CA, USA) and filtered at 1 kHz. Currents were digitized using a Digidata 1222 A board (Molecular Devices, Sunnyvale, CA). Currents were recorded from borosilicate glass electrodes (1.5 mm OD, 0.85 mm ID) pulled on a P-1000 Flaming-Brown horizontal micropipette puller (Sutter Instruments, Novato, CA, USA), using a three- stage pull to a final resistance of 5–8 mΩ. Glass electrodes were filled with an internal solution containing (in mM): 117.5 CeMeSO_4_, 1 MgCl_2_, 10 HEPES, 0.3 EGTA, 10 TEACl, 15.5 CsCl, 8 NaCl, 4 MgATP, 5 QX-314 (pH 7.5, 295–300 mOsm) and mEPSCs from each neuron were held at −70 mV throughout the recording. Series resistance and capacitance were compensated for each neuron. Input and series resistance were monitored throughout the experiment and recordings in which either changed significantly were discarded.

Spontaneous EPSCs (sEPSCs) were recorded in the presence of the GABA_A_ receptor antagonist picrotoxin (50 µM). Tetrodotoxin (TTX, 1 µM) was added to pharmacologically isolate mEPSC events. The willardine-derived KAR antagonist UBP310 (5 µM) was used to block KAR-mediated currents. Drugs were perfused for a minimum of 10 minutes to obtain a stable baseline. All chemicals were purchased from Sigma Chemical Company (St. Louis, MO) with the exception of TTX (Tocris), UBP310 (Abcam) and Picrotoxin (Abcam).

### *In vitro* oxygen-glucose deprivation protocol

Oxygen-glucose deprivation (OGD) was used as previously described as a model of *in vitro* hypoxic-ischemia^45,74^. Briefly, hippocampal slices and recordings were obtained in a standard aCSF as described above. Following a 10 minute baseline acquisition period the slices were perfused with a glucose-free aCSF containing (in mM): 126 NaCl, 2 KCl, 1.1 KH_2_PO_4_, 26 NaHCO_3_, 13 sucrose, 2 MgSO_4_, 1.5 CaCl_2_ and equilibrated with 95% N_2_/5% CO_2_. OGD was maintained for 10 min followed by reoxygenation with standard oxygenated glucose-containing aCSF for 10 minutes.

### Western blot analysis

Membrane protein expression of GluK2/3 and GluK5 subunits was determined in P7–9 C57BL/6 mice. Briefly, *in vivo* hypoxia was performed as described above and at 1-hour following exposure, hypoxia-experienced mice and littermate control mice were sacrificed for processing. Hippocampal region CA3 was microdissected and immediately placed in ice-cold standard radioimmuoprecipitation assay (RIPA) lysis buffer with the addition of 1 mM sodium orthovanadate and a protease inhibitory cocktail (Cocktail set I; Calbiochem, La Jolla, CA). The entire CA3 region from two littermate mice was pooled together (*n* = 1) in order to generate sufficient sample size. The samples were lysed and sonicated using a Sonic Dismembrator F60 (Fisher Scientific) and centrifuged for 10 min at 15,000 × g at 4 °C. Protein concentrations and western blot analysis were performed on the remaining supernatant.

Aliquots of 30 µg of protein were separated by SDS/PAGE (10%), transferred to polyvinyl difluoride (PVDF) membranes and blocked in TBS plus 0.01% Tween 20 (TBS-T) plus 5% non-fat dry milk for 60 min at room temperature. PVDF membranes were incubated with primary antisera (in TBS-T and 1% BSA) overnight at 4 °C with gentle shaking. Primary antibodies included rabbit monocloncal GluK2/3 (1:1000; Millipore) and rabbit polycloncal GluK5 (1:1000; Millipore). Following overnight incubation, membranes were washed with TBS-T and developed using enhanced chemiluminescence reagents (ECL, PerkinElmer). Normalization for protein loading was performed using a mouse monocloncal primary antibody selective for β-actin (1:2,500; Sigma). After completion of the Western blot, membranes were carefully cut lengthwise with the top portion placed into the appropriate primary antibody (GluK2/3 or GluK5) and the bottom portion placed into β-actin. To allow for optimal images and avoid over-saturation, membranes were scanned separately on a ChemiDoc Touch Imaging System (BioRad, Hercules, CA).

### Mass Spectrometry

The brain concentration of UBP310 was examined by mass spectrometry in neonatal (P9) mice. Four mice were treated subcutaneously with UBP310 (20 mg/kg) followed 30 mins later by the *in vivo* hypoxia protocol outlined above. Additionally, two naïve control mice were exposed to the *in vivo* hypoxia protocol. Following approximately 2.5 mins of reoxygenation animals were rapidly sacrificed and samples were delivered to the core facility after acetonitrile preparations. The LC-MS system consists of a Thermo Electron TSQ Quantum Access Max mass spectrometer with a HESI source interfaced to a Waters Atlantis T3 5 µm, 2.1 × 150 mm column. 10 µL of the extract was injected and the peptides eluted from the column by an acetonitrile/0.1 M acetic acid gradient at a flow rate of 400 µL/min over 0.25 hours (0.4 hours total time). The nanospray ion source was operated at 2.1 kV. The sample was analyzed by MS, MS/MS and MRM quantification. A MRM was set up to monitor the transition specific for UBP310 (354.0–197.0). The limit of detection (LOD) was ~0.5 pg while the lower limit of quantification (LLOQ) was ~2.5 pg. The standard curve over several orders of magnitude reached R^2^ = 0.998.

### Data analysis

Off-line electrophysiological data analysis was performed using Clampfit 10.3, MiniAnalysis 6.0 (SynaptoSoft, Inc., Decatur, GA) and Origin 7.5 (OriginLab, Northampton, MA). Amplitude, frequency and decay times were analyzed using a threshold for current detections at 2 times the root mean square of baseline noise. All currents were visually detected. Decay times were analyzed by fitting individual post-synaptic currents with a 10–90% rise time to a 2-exponential curve characterized by 2 time constants (τ1 and τ2) and accepted if r^2^ > 0.70. Weighted tau (Tω) was calculated using the following formula–

1 $$T\omega =\frac{{A}_{1}{T}_{1}+{A}_{2}{T}_{2}}{{A}_{1}+{A}_{2}}$$

Western blots were scanned using ChemiDoc Touch Imaging System (BioRad, Hercules, CA) and optical densitometric analysis was performed using ImageJ 1.47 v (National Institutes of Health, USA). Statistical significance was determined with the Chi-square test, Student’s *t*-test, or the non-parametric Kruskal-Wallis ANOVA for multiple groups and Tukey’s multiple comparison test for post hoc analysis as appropriate. A value of *p* < 0.05 was considered statistically significant. Values are presented as mean or percent mean ± SEM.

## Electronic supplementary material

Dataset 1
