# Supplementary material for: The Role of Kainate Receptors in the Pathophysiology of Hypoxia-Induced Seizures in the Neonatal Mouse
Source: Sci Rep. 2018 May 4;8:7035. doi: 10.1038/s41598-018-24722-3 (PMC5935682; doi:10.1038/s41598-018-24722-3)
Supplement: Supplementary file 1 — Dataset 1 [file 41598_2018_24722_MOESM1_ESM.docx]

**The Role of Kainate Receptors in the Pathophysiology of Hypoxia-Induced Seizures in the Neonatal Mouse**

**Denise K. Grosenbaugh**^a^**, Brittany M. Ross**^b^**, Pravin Wagley**^a^ **and *Santina A. Zanelli**^b^

^a^ Department of Neurology, University of Virginia, Charlottesville, Virginia, 22908

^b^ Department of Pediatrics, University of Virginia, Charlottesville, Virginia, 22908

**Corresponding Author**

Santina A. Zanelli, MD, PhD

Department of Pediatrics

P.O. Box 800386

Charlottesville, VA, 22908

Phone- 434-924-5428

Email- [sz5d@virginia.edu](mailto:sz5d@virginia.edu)

**Supplemental Figure S1**

**
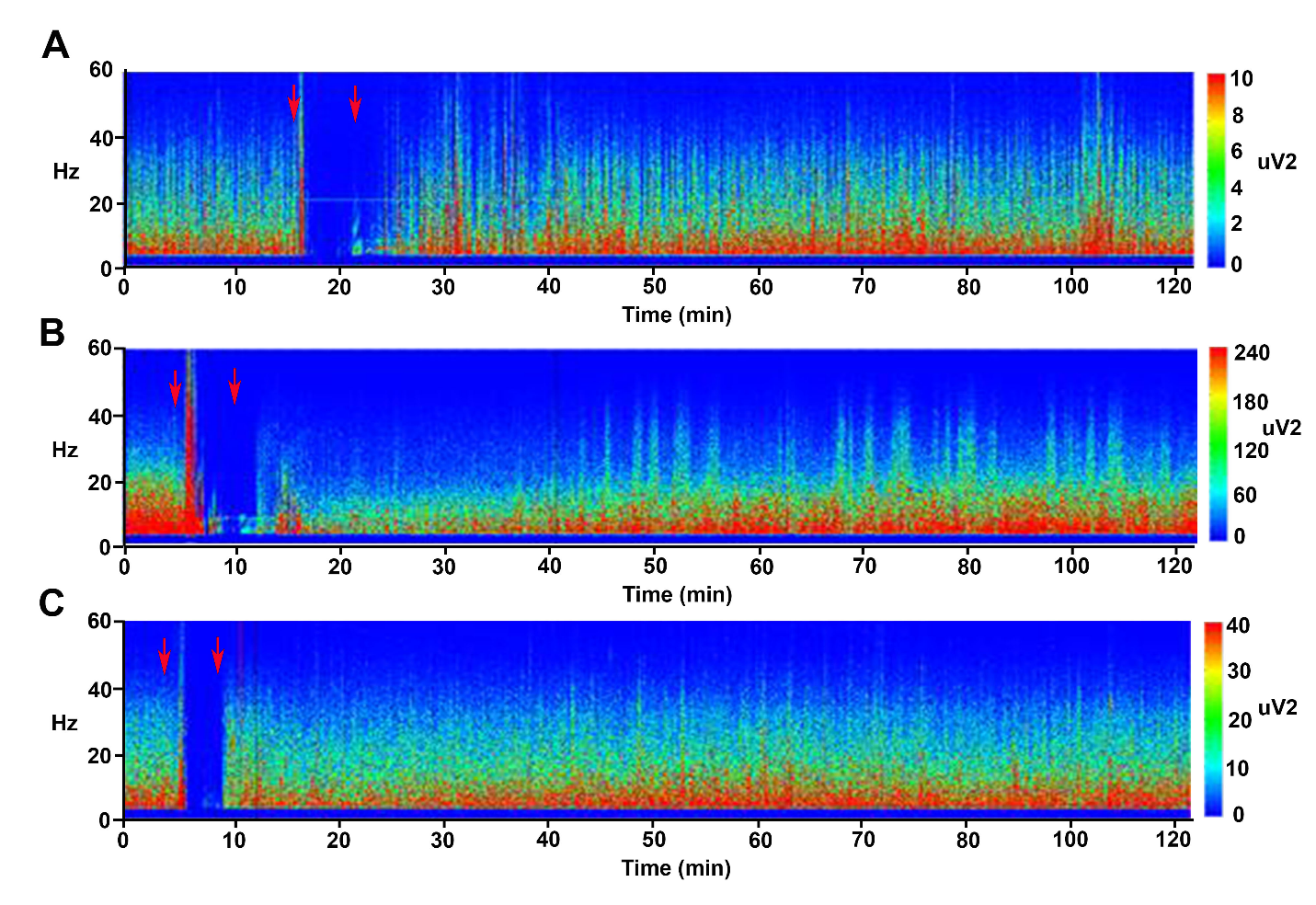
**

**Supplemental Figure S1. Effects of hypoxia and reoxygenation on total EEG power.**

EEG total power during hypoxia and reoxygenation is shown in a control (**A.**) GluK2^-/-^ (**B.**) and UBP310-treated (20 mg/kg, **C.**) neonatal mice. The impact of hypoxia with marked suppression of background EEG activity can be seen in all 3 animals followed by recovery during reoxygenation. Total power shown in μV^2^. EEG frequencies (y-axis, Hz) were filtered (0Hz high-pass, 60Hz low-pass). The red arrows indicate the onset of hypoxia and reoxygenation. All images were obtained using the spectrum view from LabChart 8.

**Supplemental Figure S2**

**
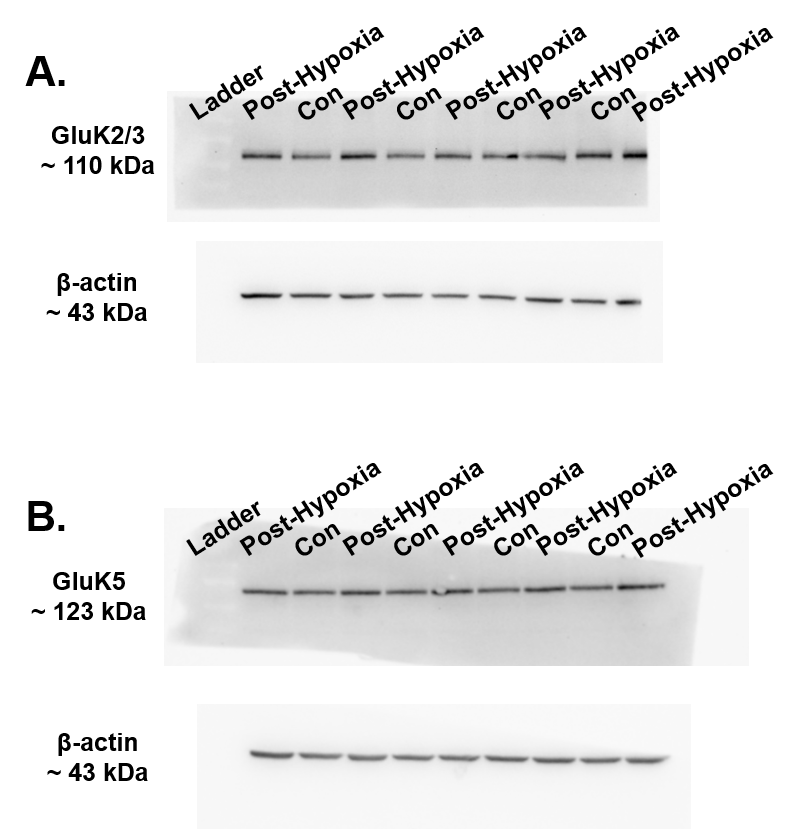
**

**Supplemental Figure S2. Full-length Western blots from area CA3 of the neonatal mouse post-hypoxia.** Full-length gel images demonstrating that expression of GluK2/3 is increased (***A.***) with no change in in GluK5 subunit expression (***B.***) in area CA3 of the neonatal mouse 1-hour post *in vivo* hypoxia. *n* = 5 – 8 pooled samples (microdissected CA3 regions from two littermate pups, *n* = 1).

**Supplemental Figure S3**

**
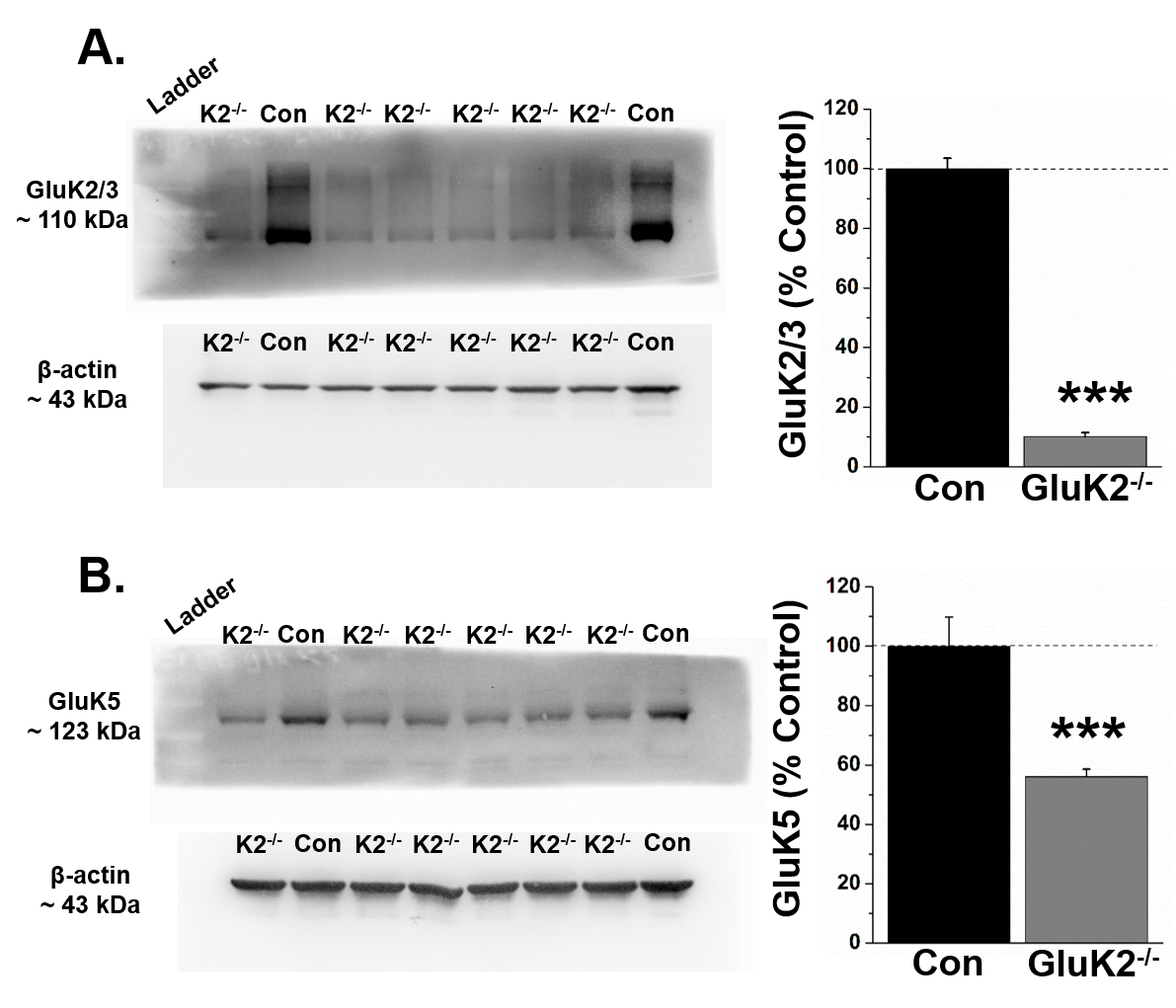
**

**Supplemental Figure S3. GluK5 subunit expression is significantly decreased in area CA3 in GluK2^-/-^ mice.** Expression of both the GluK2/3 subunit (***A.***) and the GluK5 subunit (***B.***) is significantly reduced in neonatal GluK2^-/-^ mice. *n* = 2 – 6 pooled samples (microdissected CA3 regions from two littermate pups, *n* = 1). ****p* < 0.001, *t*-test. Data shown as a percent mean ± SEM.

**Supplemental Figure S4**


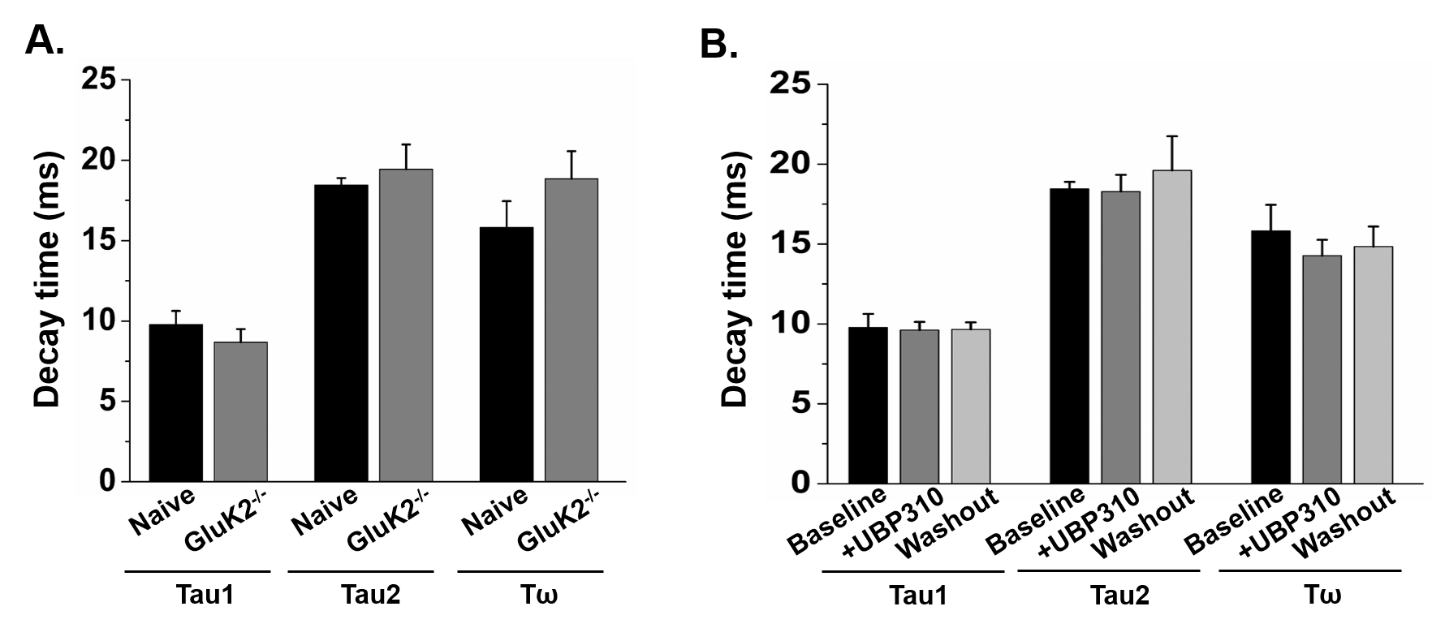


**Supplemental Figure S4. Decay kinetics were not different between experimental groups.**

Decay kinetics between naïve C57Bl/6 mice and GluK2^-/-^ were not significantly different (Tau1 – naïve: 9.78 ± 0.85 ms, GluK2^-/-^: 8.67 ± 0.82 ms, *p* = 0.383; Tau2 – naïve: 18.45 ± 0.44 ms, GluK2^-/-^: 19.44 ± 1.54 ms, *p* = 0.618; Tω – naïve: 15.82 ± 1.63 ms, GluK2^-/-^: 18.84 ± 1.71 ms, *p* = 0.247, *n* = 6 – 9 animals). Similarly, in naïve animals no difference in decay kinetics were observed during application of UBP310 (Tau1 – baseline: 9.78 ± 0.85 ms, UBP310: 9.61 ± 0.52 ms, washout: 9.66 ± 0.45 ms, *p* > 0.05, ANOVA; Tau2 – baseline: 18.45 ± 0.44 ms, UBP310: 18.29 ± 1.04 ms, washout: 19.60 ± 2.14 ms, *p* > 0.05, ANOVA; Tω – baseline: 15.82 ± 1.63 ms, UBP310: 14.27 ± 0.99 ms, washout: 14.84 ± 1.26 ms, *p* > 0.05, ANOVA; *n* = 6 animals).

**Supplemental Table S1. Seizure data from control and GluK2^-/-^ neonatal mice exposed to *in vivo* hypoxia.**

**
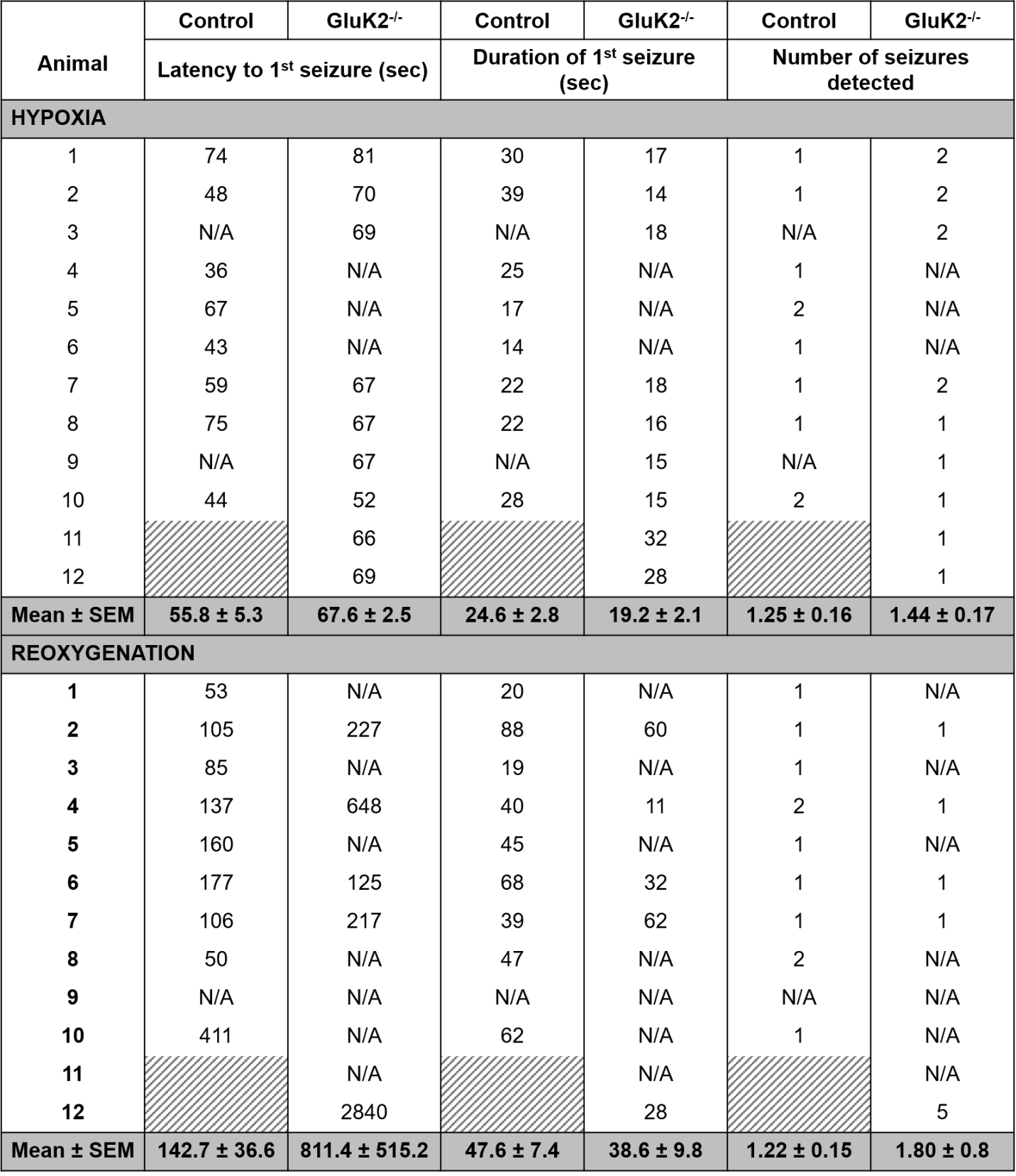
**

N/A, no seizure detected; hashed lines, no animal

**Supplemental Table S2. Seizure data from control and UBP310 pre-treated neonatal mice exposed to *in vivo* hypoxia.**

**
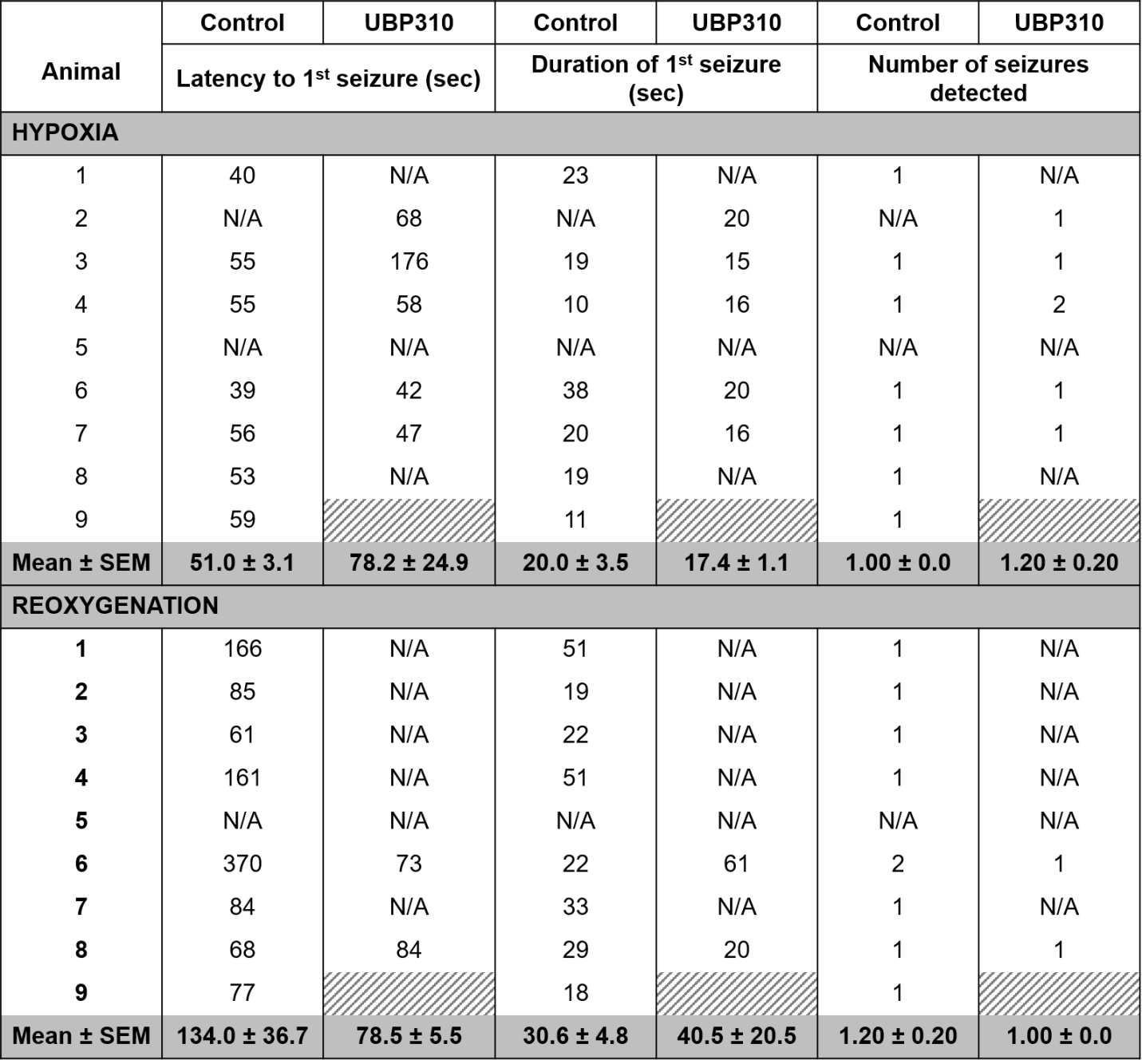
**

N/A, no seizure detected; hashed lines, no animal

**Supplemental Table S3. Brain concentration of UBP310 in neonatal mice.**

**
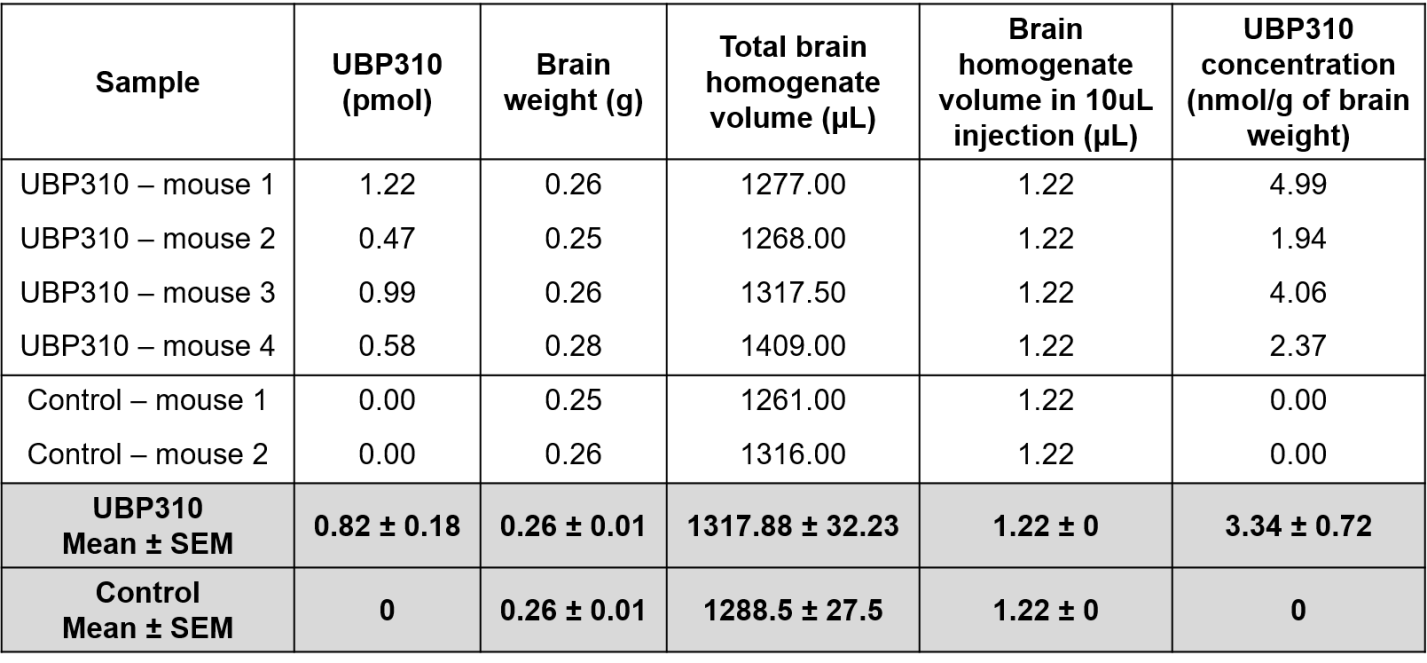
**
